# Colorimetric Systems for the Detection of Bacterial Contamination: Strategy and Applications

**DOI:** 10.3390/bios12070532

**Published:** 2022-07-16

**Authors:** Dong-Min Kim, Seung-Min Yoo

**Affiliations:** 1 Center for Applied Life Science, Hanbat National University, Daejeon 34158, Korea; dmk.iqbio@gmail.com; 2 School of Integrative Engineering, Chung-Ang University, Seoul 06974, Korea

**Keywords:** colorimetry, detection, nanomaterial, bacterial contamination, chromogen

## Abstract

Bacterial contamination is a public health concern worldwide causing enormous social and economic losses. For early diagnosis and adequate management to prevent or treat pathogen-related illnesses, extensive effort has been put into the development of pathogenic bacterial detection systems. Colorimetric sensing systems have attracted increasing attention due to their simple and single-site operation, rapid signal readout with the naked eye, ability to operate without external instruments, portability, compact design, and low cost. In this article, recent trends and advances in colorimetric systems for the detection and monitoring of bacterial contamination are reviewed. This article focuses on pathogen detection strategies and technologies based on reaction factors that affect the color change for visual readout. Reactions used in each strategy are introduced by dividing them into the following five categories: external pH change-induced pH indicator reactions, intracellular enzyme-catalyzed chromogenic reactions, enzyme-like nanoparticle (NP)-catalyzed substrate reactions, NP aggregation-based reactions, and NP accumulation-based reactions. Some recently developed colorimetric systems are introduced, and their challenges and strategies to improve the sensing performance are discussed.

## 1. Introduction

Bacteria can be found in the air, soil, and water and play central roles in ecosystems. However, bacterial contamination poses a huge threat to global public health, affecting the food industry, hospital diagnostics, and society [1,2,3,4]. According to the Centers for Disease Control and Prevention (CDC), around 48 million people suffer from diseases caused by foodborne pathogens every year. Of this, 128,000 people are hospitalized and nearly 3000 people die, resulting in billions of dollars in medical expenses and productivity loss in the United States [5]. One of the major causes of waterborne diseases is bacterial infections associated with drinking contaminated water, especially in developing countries [6,7]. Foodborne and waterborne outbreaks have increased with various factors, such as changes in the emergence of antibiotic-resistant bacteria, the adaptation of bacteria to environmental changes, changes in food processing, production, and distribution, exposure to unsafe drinking water, inadequate sanitation control systems, inadequate public health services, and increased international travel [8,9]. Given this situation, bacterial contamination should be detected and monitored to ensure environmental and food safety and reduce the incidence of bacterial infection-related diseases. There is an urgent need for simple, rapid, on-site, and sensitive methods for early diagnosis and adequate management to prevent or treat pathogen-related illnesses.

Efforts to develop an effective system have led to the development of various biosensors using novel nanomaterials and nanostructures. Biosensors, which are analytical devices, convert recognizable biological signals into directly measurable signals using various sensing methods. Sensing methods such as electrical, electrochemical, mechanical, and optical methods have attracted increasing attention due to their high sensitivity, specificity, small assay volume, and shorter detection time and have been employed for sensitive and specific detection in clinical and environmental settings [10,11,12,13]. Notably, colorimetric sensing systems may be used for the detection of bacterial contamination due to their simple and on-site operation and rapid signal readout with the naked eye that requires no external instrument or detector. This approach has also various advantages such as rapidness, non-contact detection, compact design, cost-effectiveness, portability, and complex data processing [14,15,16,17,18]. These distinct advantages have led to the recent advances in colorimetric detection strategies and technologies, which have been actively applied to detect and monitor bacterial contamination in several fields and have played a key role in fulfilling industrial and academic needs for diagnostics [14,15,16,17,18]. Therefore, it is expected that colorimetric detection systems will have increasingly important roles in point-of-care testing and monitoring pathogenic bacteria in clinical and environmental settings and the food industry.

In this article, we review the recent developments in the field of colorimetric sensing of bacterial contamination for food, water, and environmental safety and their quality control. This article focuses on pathogen detection strategies and technologies based on reactions that affect the color change for signal readout, which can be largely divided into the following five categories: (1) color change generated by external pH change-induced pH-responsive molecules (pH indicator) reactions, (2) color change generated by intracellular enzyme-catalyzed chromogenic reactions, (3) color change generated by enzyme-like nanoparticle (NP)-catalyzed substrate reactions, (4) color change by aggregated NPs, and (5) color change by concentrated NPs.

Representative applications of colorimetric detection and monitoring, including those described in this paper and others that have been developed and employed for the detection of a wide range of bacteria and toxins, are shown in Table 1. Their challenges, together with strategies to overcome the limitations, are summarized in Table 2. This review also describes an approach that can allow the development of colorimetric detection systems to facilitate their practical use.

## 2. Colorimetric Detection and Monitoring Strategies of Bacterial Contamination

Currently, the color development stage of colorimetric systems has five potential routes for signal visualization to determine the bacteria contamination level. The first route is based on external pH change-induced pH indicator reactions [19,20,21,22,23]. In this route, visual signal readouts can yield a color change in pH indicators via external pH changes. pH indicators include litmus, phenol red, bromothymol blue (BTB), and phenolphthalein. External pH changes rely on the catalytic activity of various enzymes added to the solution. These enzymes include urease [24], glucose oxidase (GOx) [25,26], esterase [27], penicillinase [28], and organophosphate paraoxon [29]. Urease, one of the most widely used enzymes, especially for the detection of bacterial contamination, hydrolyzes urea into ammonium and carbon dioxide, which increases the pH value [19,30]. Its hydrolytic activity also increases the ionic strength of the solution and, thus, can serve as a signal amplifier. Urease has been widely used in colorimetric bacterial detection based on its catalytic activity [19,20,21]. In this route, the type of pH indicator is also important, which determines the sensitivity of the system. The concentration of the indicator is another factor that determines the sensitivity and the linear range that can be quantified.

The second route relies on intracellular enzyme-catalyzed chromogenic reactions [31,32,33,34,35,36]. This route is based on the colorization of colorless or faintly colored chromogens by chemical reaction. Chromogens exhibit a variety of colors when their colorless indole molecule are substituted with halogen atoms. The degree and type of color change depend on the electron withdrawing capability of the halogen and the position of the substituted halogen on the indole ring. Depending on what kind of intracellular enzyme is used, there are two different strategies catalyzing chromogenic reactions to detect bacteria. One is to use the enzymes involved in distinctive metabolic reactions and the other is to use enzymes in the universal reactions of bacteria. In the first strategy, unique metabolic reaction-associated enzymes can not only be used as markers to detect and identify bacterial species, but also allow colorimetric bacterial detection by interacting with their chromogens. The second strategy is based on the enzymatic reaction of the metabolic pathway inherent in bacteria. This method can achieve broad-spectrum bacterial detection but may require additional processes for the identification of bacterial species. In this process, the bacteria in a sample can directly reduce the substrate, which changes the color of the substrate [31,32,33,34]. Alternatively, the presence of bacteria in a sample can act as an inhibitor of the chemogenic reaction, which leads to no color change [35,36]. This method can also monitor the antibiotic-resistant level owing to the inhibition of cell viability or metabolic reaction associated with the mode of action of antibiotics. Such a system takes advantage of the intrinsic catalytic reaction of intracellular enzymes and, thus, is easy to operate and does not require any additives except a substrate corresponding to the enzyme. Furthermore, most systems do not require any receptor to capture bacterial cells, which makes the process simpler and more cost-effective. The mixing between the substrate and bacterial cells is important because of the sensing output produced from the binding of bacterial cells to the chromogen. The degree of color change is proportional to the cell concentration. Nevertheless, the interference of physiological cellular functions by redox molecules should be further investigated.

The third route is based on enzyme-like NP-catalyzed substrate reactions [37,38,39,40,41,42,43,44]. Enzyme reactions have been widely used for the visual detection of bacteria; however, they have limited advantages due to their low stability and the high cost of enzymes. To overcome their limitations, colorimetric detection systems based on reactions using functional NPs have been developed [45,46]. An strategy of using NPs for colorimetric detection is to harness their enzyme-like catalytic activity. In comparison with natural enzymes, functional NPs have several advantages, including high stability against harsh environmental conditions (pH and temperature), proper catalytic activity, low cost for synthesis and mass production, and facile functionalization [45,47]. Enzyme-like NPs include peroxidase mimics, oxidase mimics, catalase mimics, and hydrolase mimics. In such a system, these nanomaterials act as enzymes catalyzing chromogenic reactions, allowing the visual detection of pathogenic bacteria with a fast response and simple operation even under harsh conditions.

The fourth route is based on NP aggregation reactions [48,49,50,51,52,53,54,55,56,57]. The color change is based on the size of NPs and their degree of aggregation. Among the various NPs, gold NPs (AuNPs) have been most widely used for the detection of biomolecules because of their biocompatibility and low cytotoxicity. AuNPs have also been widely used in colorimetric systems because they can change color depending on the interparticle distance through transition between dispersion and aggregation states when exposed to diverse stimuli, such as salts, ions, pH changes, chemicals, and target molecules [48,49,50,51,52,53,54,55,56,57]. Depending on the type of stimulus, several strategies can be adopted. One strategy involves the salt-induced aggregation of AuNPs [48,49,50]. High salt levels can neutralize the stabilizing electrostatic forces on AuNPs, causing the van der Waals forces to drive the conversion of dispersion into aggregation [58]. Another strategy involves the ion-induced aggregation of AuNPs [51,55,59]. For example, Mn^2+^ can interact with carboxyl groups on the AuNP surface and lead to the aggregation of AuNPs, resulting in a change in color from red to purple. A recent study reported that the addition of Mn^2+^ and Cu^2+^ facilitates the cysteine-induced aggregation of AuNPs [59]. The thiol group of cysteine strongly binds to the AuNP surface, forming a zwitterionic structure with carboxyl and ammonium groups [60]. The zwitterionic attractions of the oppositely charged groups and hydrogen bonding induce AuNP aggregation [61]. AuNP aggregation, in turn, reduces the interparticle distance between NPs and induces interparticle surface plasmon coupling, resulting in a change in color from red to blue [62]. Mn^2+^ and Cu^2+^ can form a stable chelate complex with the zwitterionic groups of cysteine molecules attached to AuNPs, thereby enhancing the colorimetric response of AuNPs. The use of functionalized ligands can also lead to AuNP aggregation in the presence of a target molecule. For example, bacteriophages targeting specific strains have been engineered to bind to both bacterial cells and AuNPs and used to detect diverse species in a colorimetric manner [63,64]. AuNP aggregation can be achieved by the direct binding of the analyte to AuNPs [55].

The fifth route is based on NP accumulation reactions [65,66,67,68,69,70,71,72,73,74,75,76,77,78,79]. This strategy has been generally used in flat substrate-based systems, such as the lateral flow assay (LFA), µ-paper-based analytical device (µ-PAD), and paper-based dipstick system [65,66,67,68,69,70,71,72,73,74,75,76,77,78,79]. These systems rely on the capillary action of fluids and colored NPs. The liquid sample containing the target molecules flows along the test device and passes through various zones of strips on which molecules that can interact with the analyte are attracted. The colorimetric readout is attributed to the conjugation of concentrated NPs with ligands targeting target molecules. The concentrated NPs act as colorimetric labels or color generation factors. Colored NPs used for colorimetric detection include AuNPs, silver NPs (AgNPs), and colloidal carbon [65,66,67,68,69,70,71,72,73,74,75,76,77,78,79]. Red AuNPs are one of the widely used colorimetric labels because of their bright color, high stability, and high biocompatibility [80]. NPs with nanozyme or peroxidase-like amplification activities are also used in this strategy, resulting in a signal amplification effect [69,70,71]. These systems based on a concentrated NP-mediated signal readout exhibit rapid responses, portability, single-step operations, cost-effectiveness, and convenience, enabling instant on-site detection and monitoring. High sensitivity, accurate quantification, and multiplexed detection remain challenges for wide application in the real world.

The aforementioned signal readout routes can be combined with diverse signal amplification methods to enhance the sensitivity of the system. Several signal amplification methods, such as hybridization chain reaction (HCR), enzymatic strand displacement amplification (SDA), loop-mediated isothermal amplification (LAMP), recombinase polymerase amplification (RPA), helicase-dependent amplification (HAD), and catalytic hairpin assembly (CHA), have been used to detect diverse bacterial cells [57,65,69,81,82,83,84]. Enzyme-driven signal amplification reactions, such as SDA and RPA, exhibit high sensitivity and specificity; however, they require relatively complicated operations, specific reaction conditions, and specific assay times depending on the catalytic ability of the enzyme. Enzyme-free isothermal amplification reactions, such as HCR, LAMP, and CHA, are low cost, have rapid responses, and require simple operations [65,83,84]. The background noise caused by non-specific products without the target should be considered. However, lower sensitivity than enzyme-driven methods remains a challenge.

## 3. Current Colorimetric Sensing Systems for the Detection of Bacteria and Toxins

In this section, we highlight the factors that affect color change for visual signal readout. The challenges and strategies to improve the performance of colorimetric sensing systems, including the related parameters (linear range, detection limit, assay time, etc.), are summarized in Table 1.

### 3.1. Color Change of pH Indicators via External pH Change

**Figure 1:**
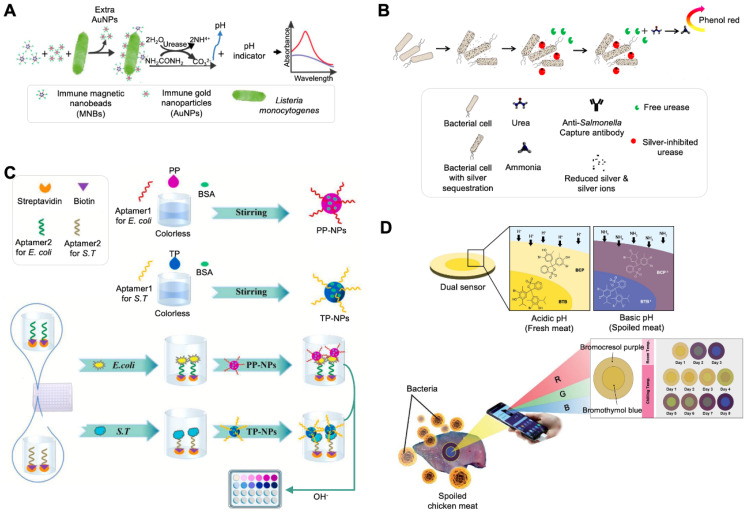
Colorimetric sensing strategy based on external pH change-induced pH indicator reactions. (**A**) Detection of Listeria monocytogenes using an antibody (Ab), urease-modified magnetic nanobeads, and gold nanoparticles (AuNPs). Reprinted with permission from [19]. Copyright 2017, Elsevier. (**B**) Detection of *Salmonella typhimurium* using silver ions and urease. This strategy is based on the Ag-induced inhibition of urease activity and Ag ion utilization. Reprinted with permission from [21]. Copyright 2020, Springer Nature. (**C**) Detection of *Escherichia coli* and *S. typhimurium* using two different pH-responsive NPs, which were made using PP or TP. Reprinted with permission from [22]. Copyright 2022, Elsevier. (**D**) Detection of bacterial spoilage using a paper-based pH indicator consisting of BTB and BCG. Quantification can be performed using RGB analysis software on a smartphone. Reprinted with permission from [23]. Copyright 2021, Royal Society of Chemistry. PEI, polyethyleneimine; BSA, bovine serum albumin; RGB, red-green-blue.

Signal readout can be obtained via the color change in a pH indicator by adding a catalytic compound that causes external pH change. Urease and a pH indicator have been widely used for the colorimetric detection of bacteria [19,20,21]. Urease is one of the most used catalytic compounds for the detection of bacterial contamination in food and the environment. To select an optimal pH indicator for yielding high sensitivity in the sensing system, three different pH indicators, phenol red, BTB, and bromocresol purple (BCP), were compared [19] (Figure 1A). After different concentrations of urea were applied, BTB exhibited the largest slope and was unaffected by urea in terms of color change. Under an optimal indicator concentration, *Listeria monocytogenes* was detected by forming magnetic nanoparticle (MNP)–monoclonal antibody (mAb)–bacterial cell–polyclonal antibody (pAb)–AuNP–urease sandwich complexes using two different NPs: MNPs for cell separation to reduce background noise and AuNPs for capturing urease via electrostatic adsorption [85,86]. The sandwich complexes containing urease could hydrolyze urea, and the increased pH of the sample could lead to a color change in BTB. The system had a detection limit of 100 CFU/mL with a linear range of 1.1 × 10^2^ CFU/mL to 1.1 × 10^6^ CFU/mL.

Considering the catalytic reaction of urease, the competitive binding ability of urease and bacterial cells to metal ions has been employed for the detection of bacteria [20,21,87]. It is well-known that urease can bind to other metals such as Ag^+^, Hg^2+^, Cu^2+^, and Zn^2+^, and metal-bound urease loses its hydrolyzing activity. Among these ions, silver ions can penetrate bacterial cell walls and exert high bactericidal activity by inactivating enzymes, inducing lipid peroxidation, and damaging cell membranes to kill bacteria. Ag ions also bind to urease, inhibiting urease activity by blocking its active site through sulfhydryl linkages that mediate a color change reaction in the pH indicator. Based on the binding ability of Ag ions to two substances, a colorimetric assay for the detection of *Salmonella* was developed (Figure 1B) [21]. In the absence of bacteria, Ag ions bind to urease, and the inhibited enzyme does not digest urea into ammonia, resulting in no pH change and no color change in the phenol red indicator. In the presence of bacteria, there is a competitive equilibrium between the enzyme and the bacteria for Ag ions. Owing to the Ag ions being sequestrated by the bacteria, the amount of free urease increases. Free urease hydrolyzes urea, and the produced ammonia leads to a higher pH, at which the phenol red indicator turns pink-red.

Similarly, the competitive binding ability of cationic AgNPs to two substances, bacterial cells and urease, has been applied to a colorimetric assay for the detection of *Salmonella*. Cationic AgNPs were fabricated by functionalizing them with polyethyleneimine, which renders the binding with the bacterial surface more attractive than that with urea because the bacterial cell surface is anionic due to an enriched negatively charged phosphate residue, and urease (pI = 5.0) is less negatively charged at a neutral pH [20]. The absence of bacteria in the sample makes AgNPs and urease bind to each other; thus, inactivated urease cannot hydrolyze urea, resulting in no pH change and a yellow color. The presence of bacteria can lead to the strong interaction of AgNPs and bacteria and can restore urease activity. Active urease produces ammonia with a color readout that allows bacterial quantification. This system is simple and cost-effective because it does not require tedious bioconjugation steps and additional receptors, and the limited identification of bacterial species can be overcome using species-specific receptors.

A recent study reported the development of pH-responsive NPs for the detection of bacterial contamination in food (Figure 1C) [22]. These pH-responsive NPs were fabricated using a phenolphthalein indicator (PP) and a thymolphthalein indicator (TP) in a self-assembly manner and functionalized by immobilizing the bacterial species-specific aptamer on each NP. The synthesized NPs were colorless at a neutral pH and red for PP-NPs and blue for TP-NPs at an alkaline pH. PP and TP also showed a rapid response to a change in the pH [88,89,90,91]. Another bacterial species-specific aptamer was modified with biotin and immobilized on a streptavidin-coated well plate. Two different aptamers captured bacterial cells present in the sample in a sandwich manner, indicating the co-existence of NPs in the sample. After NaOH was added, the increased pH caused the color change of the NPs. This process, combined with automatic equipment consisting of a pipetting head, a temperature controller, and an online control-transforming software, allowed multiplexing detection and shortened the time of the detection process to less than 1 h.

Another recent study reported the development of a paper-based colorimetric system for monitoring bacterial spoilage (Figure 1D) [23]. Bacterial spoilage changes the odor of food and increases the pH. This system relies on the pH change of food caused by volatile basic nitrogen generated from bacterial spoilage. Two different pH indicators, BCP and BTB, were coated on a piece of filter paper by soaking in a solution containing these dyes, resulting in the fabrication of a dual sensor. The dye-coated filter papers were placed onto meat and chicken fillets, and their color change was monitored for 8 days. Over time, the concentration of bacterial cells, pH value, and total basic nitrogen of the samples were increased, with the color of the paper changing from yellow to blue in the case of BTB and from yellow to purple in the case of BCP. The readout from the sensor was also analyzed using RGB software on a smartphone for the quantification of bacterial spoilage.

### 3.2. Color Change of Chemicals via Metabolic Activity of Intracellular Enzymes

**Figure 2:**
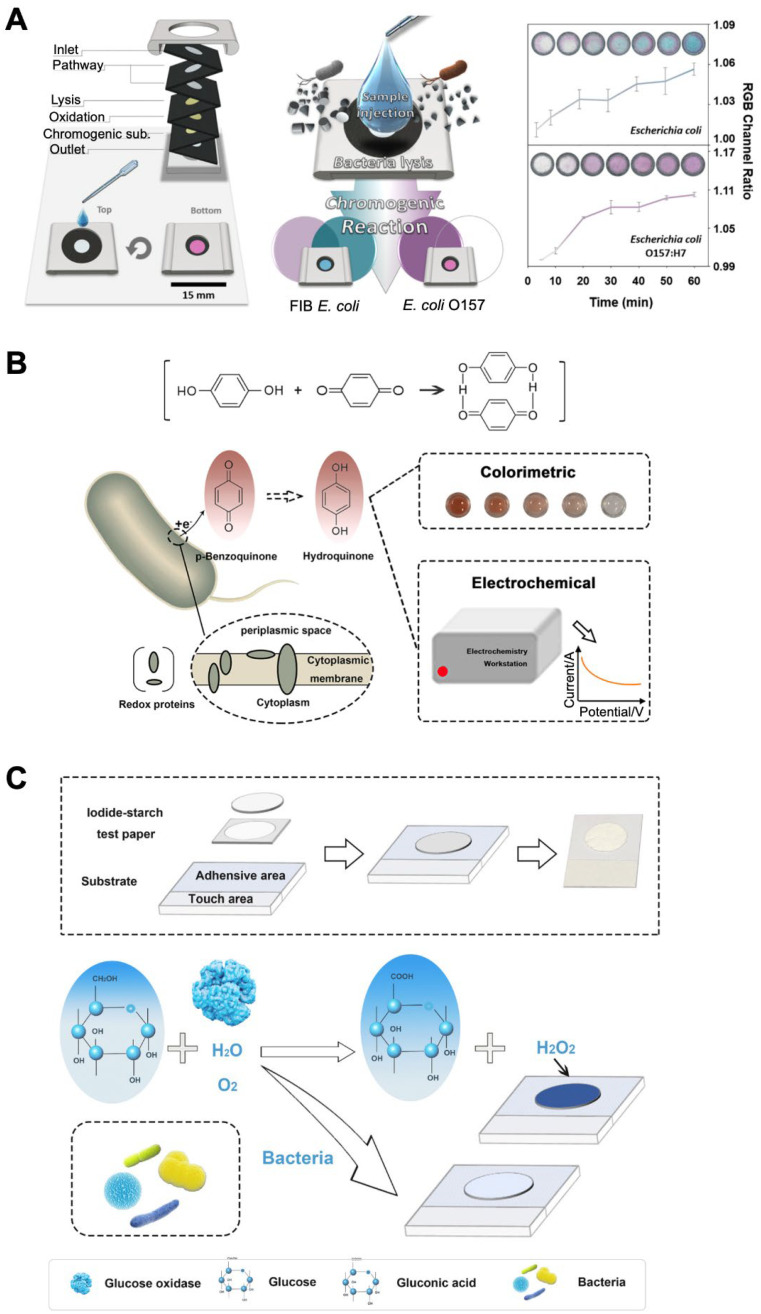
Colorimetric sensing strategy based on intracellular enzyme-catalyzed chromogenic reactions. (**A**) Discrimination of Escherichia coli and *E. coli* O157:H7 based on different cellular metabolic reactions (β-glucuronidase- and β-galactosidase-based enzymatic reactions, respectively). Reprinted with permission from [32]. Copyright 2019, American Chemical Society. (**B**) Detection of *E. coli* and *Staphylococcus aureus* based on the reduction reaction of *p*-benzoquinone by intracellular enzymes. Reprinted with permission from [34]. Copyright 2019, American Chemical Society. (**C**) Detection of *E. coli*, *S. aureus*, *Enterococcus faecalis*, *Streptococcus mutans*, and *Salmonella pullorum* based on the conversion from iodide to iodine on the starch–iodide doping paper. This conversion is catalyzed by H_2_O_2_ produced from glucose oxidase-mediated oxidation and glucose uptake of bacterial cells. Reprinted with permission from [35]. Copyright 2019, Elsevier. WST-8, 2-(2-methoxy-4-nitrophenyl)-3-(4-nitrophenyl)-5-(2,4-disulfophenyl)-2H-tetrazolium, monosodium salt; RGB, red-green-blue; FIB, fecal indicator bacteria.

Bacteria have distinctive metabolic reactions, producing substrates that are essential to the metabolism and survival of other organisms. In cellular respiration, electrons from carbon sources such as glucose move gradually through the electron transport chain toward oxygen, transitioning to lower energy states and releasing energy. The released energy is used to convert adenosine diphosphate into adenosine triphosphate, which can be used to power biological processes throughout the cell. Metabolic reactions in bacteria can trigger chromogenic reactions by directly reducing the substrate added to the solution, producing a visual readout. For example, non-O157 *Escherichia coli* has two metabolic pathways for cleaving 6-chloro-3-indoxyl-β-D-galactopyranoside (Sal-β-gal) and 5-bromo-4-chloro-3-indoxyl-β-D-glucuronic acid (X-β-gluc), producing chemicals that are purple and blue, respectively. In contrast, *E. coli* O157 only exhibits Sal-β-gal-degrading enzyme activity. Based on this discriminative feature, a system for detecting two different strains was developed with paper substrates [31,32]. The paper-based system consists of a multi-layered filter paper structure patterned with photoresists by ultraviolet light curing and functionalized with lysing, oxidizing, and chromogenic reagents in the different layers. By passing through successive layers of paper after injecting the sample, bacterial cells in the sample are lysed; the pH is adjusted for the optimal oxidation of the reactant in the oxidizing agent layer, and finally, the color is produced via the enzymatic reaction of the reactant in the chromogenic agent layer. The use of reagent-embedded paper allows the cost-effective and simple detection of bacterial species, even in resource-limited regions. This system could detect bacteria up to 10 CFU/mL with a linear range of 10^6^ to 10^8^ CFU/mL within 60 min for best visualization (Figure 2A; Table 1) [32]. With a similar system, four different bacterial species (*E. coli*, *E. coli* O157:H7, *L. monocytogenes*, and *Vibrio vulnificus*) were detected after expanding the number of chromogenic substrates to include X-β-gluc, 5-bromo-6-chloro-3-indoxyl-β-D-galactose, Aldol^®^ 518 myoinositol-1-phosphate, and 5-bromo-4-chloro-3-indoxyl-β-D-glucose, each of which could react with β-glucuronidase, β-D-galactosidase, myoinositol mono-phosphates, and β-D-glucosidase, producing sky blue, burgundy, light pink, and strong blue colors, respectively [31]. This system also had a detection limit of 10 CFU/mL.

Oxidation–reduction reactions also occur during cellular respiration in living cells. Redox-active molecules can replace oxygen in respiratory enzymatic reactions and accept electrons for living cells. Intracellular oxidoreductase reduces redox-active molecules such as organic dyes, ferricyanide, dichlorophenolindophenol, and benzoquinone, resulting in color change or decolorization. The degree of color change in these molecules induced by intracellular enzymes can be used to assess cell metabolic activity and viability. These molecules also include 3-(4,5-dimethylthiazol-2-yl)-2,5-diphenyl tetrazolium bromide (MTT) and 2-(2-methoxy-4-nitrophenyl)-3-(4-nitrophenyl)-5-(2,4-disulfophenyl)-2H-tetrazolium, a monosodium salt (WST-8). For example, WST-8 is reduced by cellular dehydrogenase, generating an orange-yellow formazan product that is soluble in the culture medium. Through the reduction of molecules by intracellular enzymes, living or dead bacterial cells have been detected [33]. Dehydrogenase-based enzymatic reactions were observed to detect viable bacteria with a linear range of 2.6 × 10^2^ to 1.16 × 10^9^ CFU/ mL for *E. coli* and 9.75 × 10^2^ to 6 × 10^9^ CFU/mL for *Staphylococcus aureus* within 2 h. Although this system can easily detect bacteria in a broad range, it requires additional steps to identify the bacterial species.

Another redox-active molecule, benzoquinone, has been employed to develop a bacterial detection system (Figure 2B) [34]. Benzoquinone, which initially appeared as a yellowish-colored crystalline, was reduced to a white-colored hydroquinone by intracellular enzymes during cell respiration. Depending on the cell concentration, the color gradient could be monitored by the naked eye, and accurate quantification could be performed using RGB analysis software on a smartphone. The developed system had a linear range of 1.0 × 10^4^ to 1.0 × 10^9^ CFU/mL. It was also used to monitor the resistance level of *E. coli* cells against antibiotics with two different modes of action (trimethoprim and erythromycin). Trimethoprim exerts bacteriostatic activity and kills bacteria by preventing them from producing folic acid for purine and DNA synthesis. Erythromycin is a bacteriostatic antibiotic that inhibits protein synthesis by binding to 50S ribosomal subunits. Within 1 h of treatment with these two antibiotics, the colors of antibiotic-susceptible and low-antibiotic-resistant bacteria remained unchanged and faded, respectively. In contrast, the color of high-antibiotic-resistant bacteria became almost colorlessness.

Unlike chromogenic reactions triggered by cellular metabolic reactions, bacteria can inhibit extracellular chromogenic reactions, causing decolorization [35,36]. Accordingly, bacterial contamination has been detected based on the chromogenic reaction of H_2_O_2_ produced from GOx-mediated oxidation and glucose uptake by bacterial cells (Figure 2C) [35]. The substrate used was embedded with starch and iodide. After glucose was dropped on the starch–iodide doping paper in the absence of bacteria, GOx-catalyzed glucose oxidation produced H_2_O_2_, converting iodide to iodine. The produced iodine reacted with the starch, and the color of the paper changed to deep blue. When the sample was contaminated with bacteria, bacteria absorbed glucose as a carbon source for survival, inhibiting the subsequent chemical reaction and keeping the paper colorless. Using this system, *E. coli* and *S. aureus* could be detected at a concentration of 7.48 × 10^3^ CFU/mL and 3.3 × 10^3^ CFU/mL, respectively, within 20 min.

Another system based on the inhibition of chromogenic reactions by metabolic reactions has been developed using Cu^2+^ and o-phenylenediamine (OPD) [36]. Cu^2+^ can not only react with OPD, which produces oxidized OPD with a pale-yellow color, but can also bind to bacterial cells, which reduces Cu^2+^ to Cu^+^ through intracellular Cu^2+^ metabolic reaction-related enzymes [92,93]. The paper was prepared by dropping OPD solutions as a substrate. When Cu^2+^ was applied to OPD-embedded paper with no bacteria, the reaction between OPD and Cu^2+^ occurred without any inhibiting factors, changing the color of the paper to pale-yellow. However, the presence of bacteria in the sample impeded OPD oxidation by Cu^2+^, resulting in the OPD remaining colorless. Furthermore, the Cu^2+^-oxidized OPD is an orange-yellow fluorescent chemical; thus, its use could allow fluorescent and colorimetric dual-channel detection. In the dual-readout mode, this system had a detection limit of 44 CFU/mL with a linear range of 1 × 10^2^ to 1 × 10^6^ CFU/mL.

### 3.3. Color Change of Substrates via Enzyme-like Catalytic Reactions of NPs

**Figure 3:**
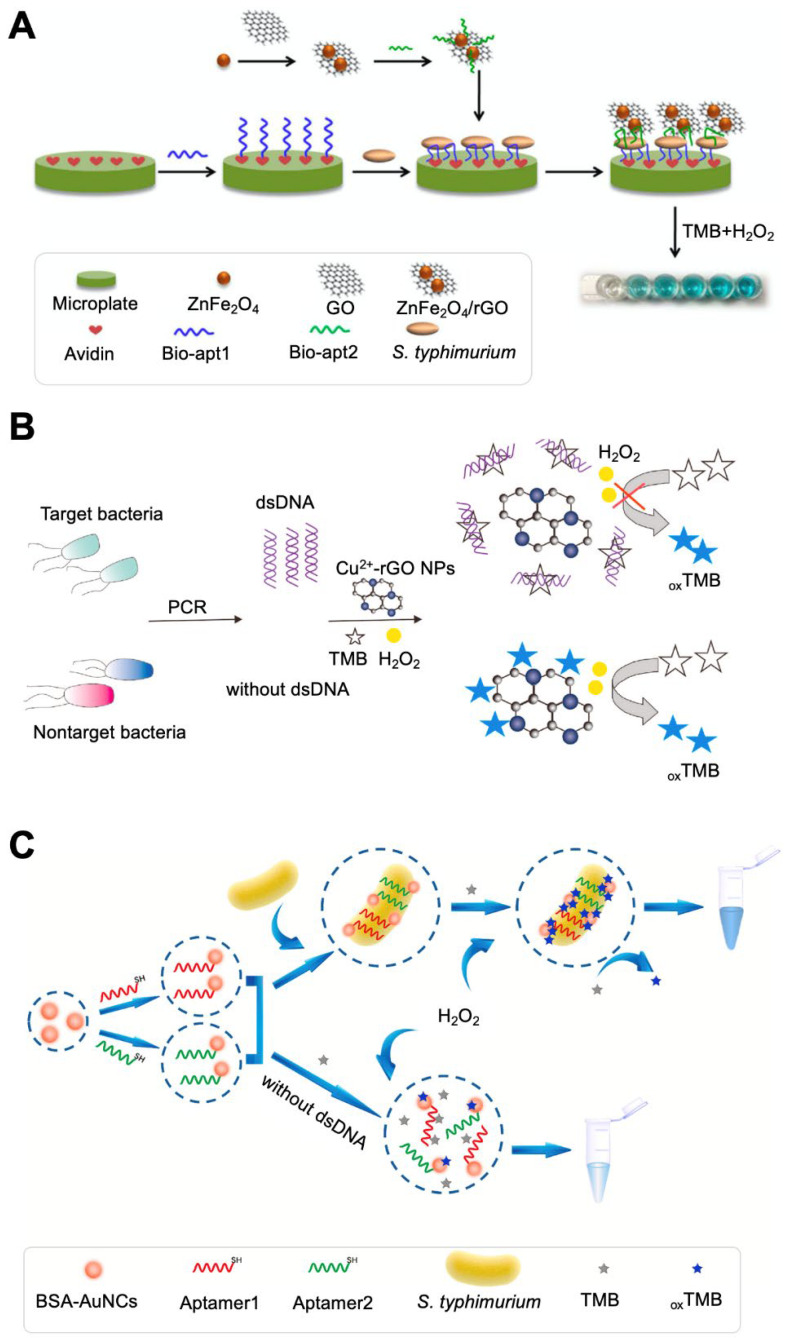
Colorimetric sensing strategy based on enzyme-like NP-catalyzed substrate reactions. (**A**) Detection of *Salmonella typhimurium* based on the peroxidase-like catalytic reaction of a ZnFe_2_O_4_/rGO nanostructure. Reprinted with permission from [37]. Copyright 2017, Elsevier. (**B**) Detection of *Salmonella* spp. based on the peroxidase-like catalytic reaction of rGO. Reprinted with permission from [38]. Copyright 2020, Elsevier. (**C**) Detection of *S. typhimurium* using AuNCs. Reprinted with permission from [43]. Copyright 2020, Elsevier. TMB, 5,5′-tetramethylbenzidine; oxTMB, oxidized TMB; AuNC, gold nanocrystal; rGO, reduced graphene oxide; PCR, polymerase chain reaction; apt, aptamer.

Pathogenic bacteria can be detected based on the color change of chromogens. The change in the visible color is attributed to the catalytic reaction between enzymes and chromogenic substrates. 3,3′,5,5′-Tetramethylbenzidine (TMB) is one of the most widely used chromogens [94,95]. It is oxidized by peroxidase in the presence of H_2_O_2_ and undergoes color change from colorlessness to blue. Such a color change can occur due to enzymatic reactions involving horseradish peroxidase (HRP) and GOx. However, enzymes have some limitations, such as low stability under harsh conditions (pH and temperature), difficulty in labeling and functionalization, and high purification cost, especially in vitro. The use of enzyme mimics could be an alternative approach to overcome the limitations of natural enzymes [14,46]. Functional nanomaterials have been widely used in the colorimetric detection of bacterial contamination because of their unique properties. The catalytic activity of some nanomaterials is similar to that of enzymes, which triggers a catalytic reaction that induces a color change in the chromogen [38,40,96]. These enzyme-like materials include Fe_3_O_4_, metallic oxide NPs (Co_3_O_4_ and CeO_2_), noble metal NPs (AuNPs, Ag/PtNPs, and Au/PdNPs), and carbon-based nanomaterials (e.g., graphene oxide (GO)) [26,37,38,39,97,98,99,100,101]. A ZnFe_2_O_4_-modified reduced GO (rGO) nanostructure with HRP-like activity was used to detect S. typhimurium (Figure 3A) [37]. *S. typhimurium* was captured by two different aptamer-sandwich complexes, and the ZnFe_2_O_4_/rGO nanostructure could bind to the aptamer due to the specific affinity of GO for single-stranded DNA. The ZnFe_2_O_4_/rGO nanostructure was highly stable, even at pH 5.5 and 50 °C, demonstrating sensitive detection with a detection limit of 11 CFU/mL.

Peroxidase-like Cu^2+^-rGO was also used for the detection of *S. typhimurium* (Figure 3B) [38]. In this study, dsDNA amplified from bacterial cells acted as a competitor of Cu^2+^-rGO in the TMB interaction. If bacteria were present in the sample, dsDNA could be extracted from bacterial cells and amplified via polymerase chain reaction, and the dsDNA could bind to TMB. The competitive binding of TMB with dsDNA hindered the interaction of Cu^2+^-rGO and TMB, resulting in the solution color remaining the same. In a bacteria-free sample, Cu^2+^-rGO-based TMB oxidation occurred in H_2_O_2_, producing a blue color. This system had a detection limit of 0.51 CFU/mL with a linear range of 1.93 × 10^1^ to 1.93 × 10^5^ CFU/mL. Another peroxidase mimic, graphitic C_3_N_4_@Cu_2_O (g-C_3_N_4_@Cu_2_O), was used for the detection of *S. typhimurium* [39]. A *S. typhimurium*-specific aptamer was attached to C_3_N_4_@Cu_2_O, and the presence/absence of cells was monitored by detecting a color change. The g-C_3_N_4_@Cu_2_O nanostructure exhibits peroxidase-like activity and its functionalization with an aptamer lowers the peroxidase-like activity of the g-C_3_N_4_@Cu_2_O nanostructure. Therefore, the presence of bacteria in the sample strengthens the change in TMB color because the binding of the aptamer and cells induced the conformational change of the aptamer and weakened the interaction of the aptamer and C_3_N_4_@Cu_2_O. This system had a detection limit of 15 CFU/mL with a linear range of 1.5 × 10^1^ to 1.5 × 10^5^ CFU/mL. The paper-based system could detect bacterial cells within 6 min.

The bioconjugation of target-specific receptors can shield the surface of nanomaterials, reducing their catalytic activity. Based on this unique characteristic, a method of detecting *S. aureus* has been developed [40]. Bacterial cells were first captured by IgY-Fe_3_O_4_/Au nanocomposites and aptamer-functionalized AuNPs in a sandwich manner. Although Fe_3_O_4_ NPs are generally used for sample separation, they have limited active sites for conjugating bioreceptors directly and can aggregate, thereby losing their magnetic properties. To overcome this limitation, MNPs covered with an Au shell were used to enhance the modification yield and stability. AuNPs behave similarly to peroxidase; however, aptamer functionalization for capturing cells on the surface of the AuNPs causes them to lose their enzymatic activity. After cells were captured, the use of a magnet allowed the separation of cell-bound nanomaterial complexes from unbound aptamer-functionalized AuNPs. The H_2_O_2_ added to unbound AuNPs dissociated the aptamer and restored the catalytic activity of AuNPs, which catalyzed TMB, producing a yellow color. This system had a detection limit of 10 CFU/mL with a linear range of 10^1^ to 10^6^ CFU/mL.

The detectable sensitivity of peroxidase activity-based TMB signals is limited because H_2_O_2_ can produce only one colored TMB molecule with the aid of peroxidase. The selection and optimization of enzyme–chromogenic substrates are important for developing advanced colorimetric assays with high sensitivity. For example, the addition of Fe^2+^ and Cu^2+^ to reactions with H_2_O_2_ and TMB can produce two colored TMB molecules by forming Fe^3+^/Cu^2+^ and hydroxyl radicals, both of which directly oxidize TMB [102,103,104,105]. Through this signal amplification strategy, brevetoxin B (BTB), a potent neurotoxin naturally produced from Karenia brevis, was detected [41]. BTB was conjugated with magnetic beads and incubated with Ab-AuNP-GOx composites. If the sample contained BTB, a Mb-BTB-Ab-AuNP-GOx complex was formed and isolated after the magnetic separation process. Upon exposure to glucose and Fe^2+^, GOx oxidized glucose to gluconic acid and H_2_O_2_. H_2_O_2_ oxidized Fe^2+^ to Fe^3+^ and hydroxyl radicals, both of which changed the color of TMB to yellow. If BTB was absent in the sample, the TMB remained colorless TMB. This system could detect up to 0.076 ng/kg of BTB.

Based on the chemical conversion of Fe^2+^ to Fe^3+^ by GOx-catalyzed H_2_O_2_, K_3_[Fe(CN)_6_] was employed as a color change indicator for visual readout [42]. K_3_[Fe(CN)_6_] is blue in the presence of a Fe^2+^-containing solution; however, the oxidization of Fe^2+^ to Fe^3+^ inhibits the production of blue color, making the solution colorless. This feature was exploited to determine the ochratoxin (OTA) contamination level [42]. OTA attached to MB was bound with OTA antibody-functionalized AuNPs, in which GOx was modified, forming the sensing platform (MB-OTA-OTA Ab-AuNP-GOx). Upon the addition of a sample, magnet-based separation was accomplished, and glucose, FeCl_2_, and K_3_[Fe(CN)_6_] were sequentially added to the precipitate. This assay is based on the competition of OTA attached to a sensing platform and OTA in a sample for binding with an anti-OTA antibody; thus, if the sample contained OTA, OTA antibody-AuNP-GOx in the sensing platform could bind with the OTA in the sample instead of that in the sensing platform. The amount of OTA antibody-AuNP-GOx in the sensing platform was decreased compared with that in the initial stage, which also led to a decreased amount of GOx, resulting in GOx-catalyzed H_2_O_2_ not being produced and, thus, K_3_[Fe(CN)_6_] not changing color. When OTA was absent from the sample, there was no competition, and the amount of OTA antibody-AuNP-GOx in the sensing platform remained the same, resulting in the production of GOx-catalyzed H_2_O_2_ and, thus, the color of K_3_[Fe(CN)_6_] changing from blue to colorless. Through this strategy, OTA could be detected up to 8.3 pg/mL with a linear range of 0.01 to 10 ng/mL.

Au nanoclusters (AuNCs) can also catalyze the oxidation of TMB by H_2_O_2_ and have been used for the detection of *S. typhimurium* (Figure 3C) [43]. AuNCs were functionalized as bacteria-specific aptamers and added to a sample containing TMB and H_2_O_2_. If the sample contained bacterial cells, bacteria could bind with not only aptamer@AuNCs but also TMB. These interactions might be possible due to the shorter distance between aptamer@AuNCs and the TMB substrate, resulting in enhanced peroxidase-like activity toward TMB. The enhanced activity may be related to the sensitivity and specificity of the system, which had a detection limit of 1 CFU/mL with a linear range of 10^1^ to 10^6^ CFU/mL.

Most peroxidase-like nanostructures are developed with a focus on catalytic activity in the presence of H_2_O_2_ as an oxidant. H_2_O_2_ is toxic to target analytes or cells and is unstable; thus, strict time control may be necessary. A MnO_2_-doped nanostructure was developed for the H_2_O_2_-free oxidization reaction of TMB into a blue-colored product [44,106]. MnO_2_-doped NPs have intrinsic peroxidase- and oxidase-like activities owing to the charge transfer complex of the iron oxide NPs, which could mediate the one-electron oxidation of TMB. MnO_2_-doped NPs could bind with bacteria via van der Waals interactions between the residual amino acids of the NPs and the anionic cell surface of the bacteria. These interactions could lead to the blocking of the active catalytic sites of NPs, causing them to lose their catalytic activity. Two different bacteria, *S. aureus* and *Vibrio parahaemolyticus*, were detected using MnO_2_-doped Fe_3_O_4_ NPs [44]. These NPs acted as multifunctional NPs, facilitating the recognition, absorption, and separation of bacterial cells as well as enzyme-like activity. This system could detect bacterial cells to 10^2^ CFU/mL with a linear range of 10^1^ to 10^6^ CFU/mL.

### 3.4. Color Change via NP Aggregation-Based Reaction

Signal readout can be observed through a change in color resulting from the aggregation of NPs, such as AgNPs and AuNPs. The color change is attributed to the reduction of interparticle distance, which induces surface plasmon coupling. Au has been widely used for the colorimetric detection of bacteria through the aggregation of NPs induced by diverse stimuli. Among various NP aggregation factors, salt-indued NP aggregation was used to detect bacterial cells in drinking water [48]. AuNPs were initially functionalized with 4-mercaptophenylboronic acid (4-MPBA), which binds to glycoprotein on the surface of bacterial cells through reversible esterification [107]. In the absence of bacteria, NaCl treatment can change the color of AuNPs from red to purple as a result of aggregation. When the sample contains *E. coli* cells, surface colloidal AuNPs are covered with bacteria by covalent bonding between 4-MPBA and saccharides; this inhibits the aggregation of AuNPs and maintains their intrinsic color. As 4-MPBA has an affinity towards saccharides and glycosylated biomolecules, it can bind to both peptidoglycans on the gram-positive bacterial cell surface and lipopolysaccharides on the gram-negative bacterial cell surface, enabling broad-spectrum bacterial detection in practical applications.

The detection of specific strains can be achieved using species-specific receptors, such as aptamers, and attaching them to AuNPs [49]. As ssDNA aptamers have a flexible phosphate backbone, the bases can easily access AuNPs and get adsorbed onto the surface of negatively charged AuNPs through van der Waals interactions. The adsorbed aptamers can act as stabilizers of AuNPs to disperse uniformly without any aggregation in high-salt solutions, thereby maintaining their red color. When aptamers that bind to bacterial cells rather than NPs are used as receptors, the presence of bacteria in samples causes the detachment of aptamers from AuNPs, resulting in the aggregation of AuNPs and the change in color from red to purple. Using this method, as low as 80 CFU/mL of *S. flexneri* could be detected within 20 min, with the linear range being 1 × 10^2^ to 1 × 10^6^ CFU/mL [49].

**Figure 4:**
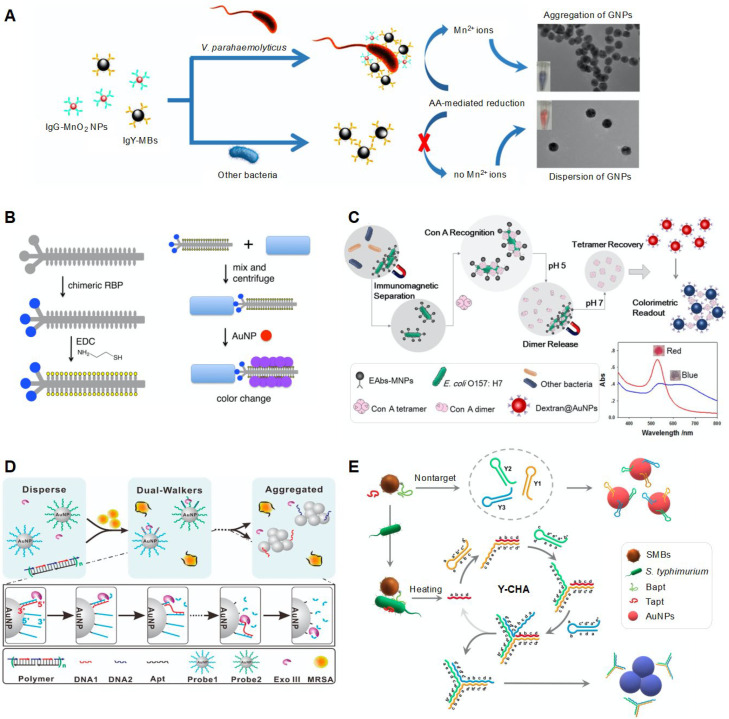
Colorimetric sensing strategy based on the aggregation of NPs. (**A**) Detection of *Vibrio parahemolyticus* based on Mn^2+^-mediated aggregation of AuNPs. Reprinted with permission from [51]. Copyright 2018, American Chemical Society. (**B**) Detection of *Escherichia coli*, *Vibrio cholerae*, *Pseudomonas aeruginosa,* and *Xanthomonas campestris* based on engineered bacteriophage-mediated aggregation of AuNPs. Reprinted with permission from [52]. Copyright 2018, American Chemical Society. (**C**) Detection of *E. coli* O157:H7 through pH-responsive concanavalin A-mediated aggregation of AuNPs. Reprinted with permission from [54]. Copyright 2017, Springer Nature. (**D**) Detection of *Staphylococcus aureus* using DNA dual-walker signal amplification combined with colorimetric systems. Reprinted with permission from [56]. Copyright 2020, American Chemical Society. (**E**) Detection of *Salmonella typhimurium* using colorimetric systems in combination with the aptamer-catalytic hairpin assembly (CHA) method. Reprinted with permission from [57]. Copyright 2021, MDPI. GNP, gold nanoparticle; AA, ascorbic acid; RBP, receptor-binding protein; EDC, 1-ethyl-3-(3-dimethylaminopropyl)-carbodiimide; ConA, concanavalin A; MNP, magnetic nanoparticle; ExoIII, exonuclease III; MRSA, methicillin-resistant *S. aureus*; SMB, streptavidin-coated MNP; Bapt, biotinylated aptamer; Tapt, non-labelled aptamer; Y-CHA, Y-shaped catalytic hairpin assembly.

Mn^2+^, which is another NP aggregation factor, can also change the interparticle distance of AuNPs and aggregate them, changing the color of the colloidal solution (Figure 4A) [51]. For the detection of *V. parahaemolyticus*, species-specific antibodies were attached to two different particles in a previous study: (1) MnO_2_ NPs for inducing AuNP aggregation and (2) MNPs for the easy separation of target analytes [51]. After *V. parahaemolyticus* was captured in a sandwich-type reaction of these particles, the added ascorbic acid reduced MnO_2_ NPs, producing MnO_2_ ions. The generated MnO_2_ ions could interact with carboxyl groups on the AuNP surface, inducing AuNP aggregation. As one bacterial cell can bind to many MnO_2_ NPs, the subsequent ascorbic acid-induced reduction of MnO_2_ NPs could facilitate Mn^2+^-induced AuNP aggregation, enhancing the sensitivity of the assay through signal amplification. Using this system, as low as 10 CFU/mL of *V. parahaemolyticus* could be detected, with the linear range being 1 × 10^1^ to 1 × 10^6^ CFU/mL.

A diverse pathogen-detecting system was developed using bacteriophages (Figure 4B) [52]. The bacteriophage acted as a ligand for binding to specific species and an NP aggregation factor. The ligand for binding to specific species was fabricated using M13 as a phage scaffold. The M13 phage is a non-lytic filamentous phage. The M13 scaffold was functionalized by displaying receptor-binding proteins (RBPs) fused to the minor coat protein pIII. Ligand specificity was achieved by engineering to displace RBPs from the M13 phage targeting *E. coli* (F^+^) into different RBPs from the following foreign phages targeting specific species: the CTXφ phage targeting *V. cholerae*, Pf1 phage targeting *Pseudomonas aeruginosa*, φLf and φXv phages targeting *Xanthomonas campestris,* and Ιφ1 phage targeting *E. coli* (I^+^). This was possible because each RBP could interact with a specific host and needed machinery for downstream infection and propagation, which was non-compatible with other hosts. The capsids of the scaffold were also chemically modified with thiol groups to bind to AuNPs, providing a signal amplification effect. When such an engineered M13 scaffold was incubated with bacterial cells present in the sample, it could capture cells and form cell–phage complexes. After centrifugation, the cell–phage complexes were separated and the added AuNPs were attached to the capsids of the M13 scaffold, inducing AuNP aggregation and a change in color to purple, as visible to the naked eye. This system had a detection limit of 10^2^ CFU/mL within 1 h. Using this engineered phage scaffold, the antibiotic resistance and susceptibility of *P. aeruginosa* could be assessed [53]. A Pf1 protein-anchored thiolated phage was engineered for *P. aeruginosa*. When the sample contained antibiotic-resistant *P. aeruginosa*, live cells bound to the engineered phage and formed cell–phage complexes. Centrifugation separated the complexes, and the incubation of AuNPs induced a color change in the solution. Antibiotic-susceptible cells are dead or have low functionality, resulting in no interaction with or low affinity to the engineered phage. After centrifugation, no or few phages remained in the solution, resulting in no or slight color change.

Concanavalin A (ConA) lectin, which is another driving factor for AuNP aggregation, was also used to detect *E. coli* (Figure 4C) [54]. ConA is a carbohydrate-binding protein that specifically binds to glycans with α-mannose-containing cores, e.g., many *N*-glycans, and has a strong affinity to the glycosyl protein on the bacterial surface. ConA has a pH-responsive tetramer–dimer transition ability; it forms tetramers at pH levels higher than 6.0 and dissociates to dimers or monomers under pH 5.0. To detect *E. coli*, antibody-functionalized MNPs were used. Bacteria in the sample could be captured and separated by MNPs. The added ConA sequentially bound to the bacterial cell surface. Low pH (pH 5.0) caused the ConA tetramers to dissociate to dimers or monomers. After removing bacteria-captured MNPs using a magnet, the dissociated dimers were transformed into tetramers with an increase in pH. The recovered ConA tetramers could interact with dextran molecules coated on AuNPs, leading to AuNP aggregation and providing a colorimetric readout. The use of ConA resulted in a signal amplification effect because of the high abundance of ConA-binding sites in bacteria. The proposed system exhibited a detection limit of 41 CFU/mL within 95 min.

Direct binding of target molecules to functionalized AuNPs can aggregate AuNPs [55]. Using this approach, the fumonisin B1 (FB1) detection method was developed because the hydroxyl groups of FB1 bind to cysteamine of AuNPs through multiple hydrogen bonds and aggregate them [55]. FB1 is a type of mycotoxin secreted from *Fusarium* species, which are mainly found in corns [108,109,110]. To detect FB1, cysteamine-functionalized AuNPs (Cys-AuNPs) were used for stabilizing NPs through strong electrostatic repulsion between particles and for facilitating interaction with FB1. The large size of FB1 causes steric hindrance; therefore, FB1 was hydrolyzed to form a structure with a short chain, which has a high affinity towards cysteamine-attached AuNPs. On hydrolyzing FB1 with KOH, it bound to dispersed Cys-AuNPs, forming aggregated Cys-AuNPs and leading to a change in color from red to purple.

The signal readout induced by AuNP aggregation can be amplified by combining diverse methods [56,57]. For example, the enzyme-driven DNA walker system was combined with the AuNP-based colorimetric method for the detection of bacterial cells (Figure 4D) [56]. The DNA walker system consisted of AuNPs functionalized with two different thiolated ssDNAs (probe 1 and probe 2, dual walkers) and DNA polymers. DNA polymers contained bacterial species-specific aptamers and walking strands (DNA 1 and DNA 2) with a sequence complementary to probe 1 and probe 2. If target bacterial cells were present in the sample, the aptamers preferred to bind to bacterial cells, thereby releasing them from the DNA polymers. Walking strands were also dispersed freely in the solution and bound to probe 1 and probe 2 on AuNPs, forming duplexes. Upon exposure to exonuclease III that could specifically digest one strand of duplex DNA from the 3′-hydroxyl termini, only probe 1 and probe 2 were removed and the surviving walking strands bound to another probe 1 and probe 2, resulting in multiple cycling reactions of hybridization and digestion. Probe DNA-removed AuNPs were eventually aggregated, and their color changed from red to purple. The color change increased with an increase in the number of walkers on AuNPs and walking strands. Using this system, as low as 1 CFU/mL of *S. aureus* could be detected within 15 min.

CHA, another signal amplification method, was used for the detection of *S. typhimurium* (Figure 4E) [57]. The CHA method relies on self-assembly and disassembly reactions between DNAs with hairpin structures. This method is an enzyme-free isothermal amplification method with high catalytic efficiency, low background signals, and simple operations in comparison with the enzyme-driven signal amplification method, which requires complex operations, specific reaction conditions, and specific assay times depending on the catalytic abilities of the enzymes [83,84]. To amplify the signal readout, three different DNAs (Y1, Y2, and Y3) were used; they formed a hairpin structure because of their low stability in terms of kinetics [57]. If *S. aureus* was absent in the sample, these DNAs were absorbed on the surface of AuNPs, preventing AuNP aggregation. If the sample contained *S. aureus*, the bacterial cells were captured by two different aptamers, namely a biotinylated aptamer (Bapt) attached to streptavidin-coated MNPs and a free aptamer (Tapt), in a sandwich manner, forming aptamer-cell-aptamer-MNP complexes. Upon heating, Tapt was released from the complexes and joined the CHA reaction by sequentially binding to three different DNAs absorbed on AuNPs, eventually forming a Y-shaped structure. Thus, AuNPs were aggregated, inducing a change in color from red to purple.

### 3.5. Color Change via NP Accumulation-Based Reaction

**Figure 5:**
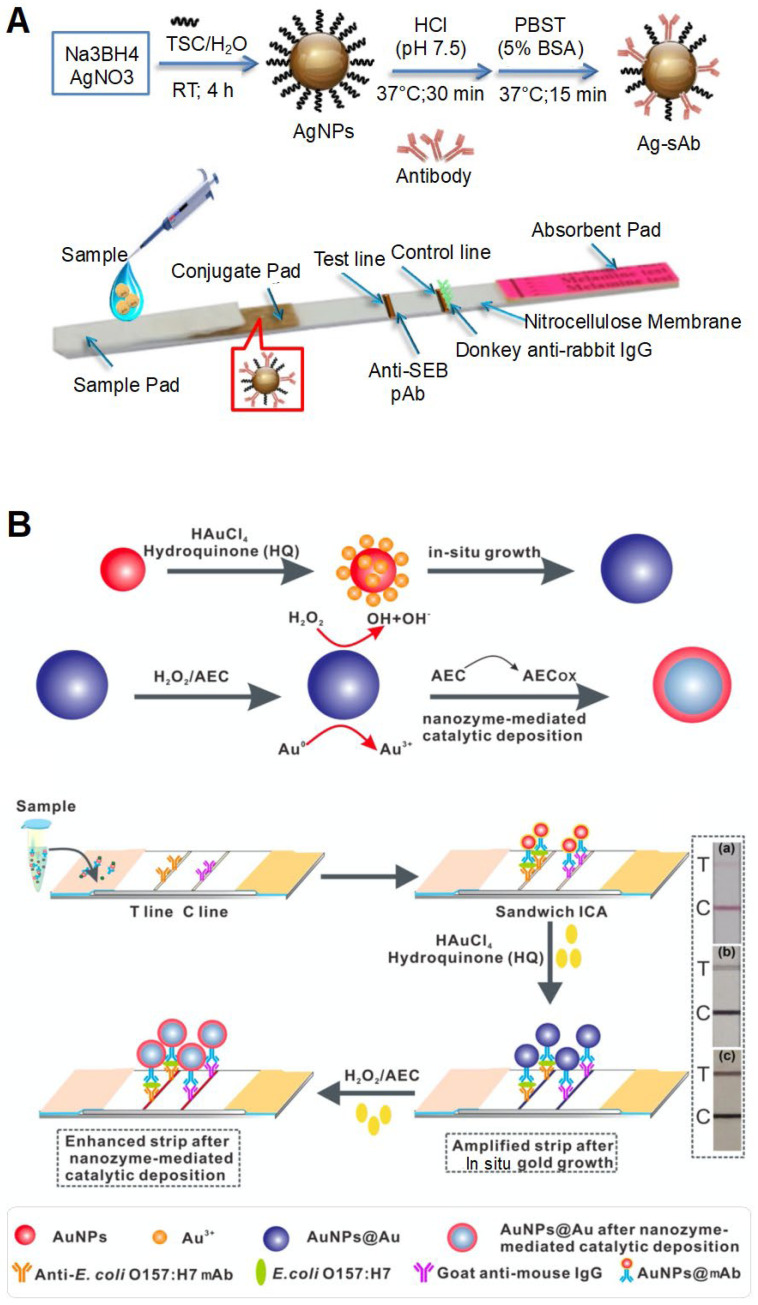
Colorimetric sensing strategy based on the accumulation of NPs. (**A**) Detection of staphylococcal enterotoxin B based on color readout by concentrated AgNPs on the lateral flow immunoassay (LFIA) strip. Reprinted with permission from [65]. Copyright 2020, Elsevier. (**B**) Detection of *Escherichia coli* O157:H7 based on color readout by concentrated AuNPs on the LFIA strip. This system consisted of a combination of two methods for cascade signal amplification. Reprinted with permission from [70]. Copyright 2020, American Chemical Society. AEC, 3-amino-9-ethylcarbazole; ICA, immunochromatography assay; SEB, *staphylococcal* enterotoxin B; mAB, monoclonal antibody.

The concentrated NPs themselves act as colorimetric labels and generated color in the test zone. The representative colored NPs widely used in this approach are colloidal AuNPs. In previous studies, AuNPs were generally applied to paper-based flow assay strips [74,75,76,111]. The concentrated NPs acted as colorimetric labels and generated color in the test zone. For example, to detect *Brucella* species, an LFA strip was fabricated in laminates consisting of a nitrocellulose (NC) membrane, conjugation pad, sample pad, and absorption pad. One anti-*Brucella* antibody was deposited on the control and test lines of the strip, and another anti-*Brucella* antibody conjugated with AuNP (Ab-AuNP) was deposited on the conjugate pad [74]. When a sample containing the target bacteria was deposited on the sample pad, the sample bacterial suspension bound to Ab-AuNPs during flow through the strip. Following this, the bacteria-antibody-AuNP complexes were captured by antibodies on the control and test lines in a sandwich manner, resulting in the accumulation of AuNPs on these lines and the appearance of intense red lines. The AuNP-based dual-antibody sandwich assay was modified in a vertical flow format. Unlike LFA, this format was designed to enable the fluid to flow vertically and pass through the pads [111]. This format consisted of two parts: the upper cover body contained staphylococcal protein A (SPA)-AuNPs and the detection cassette contained an IgG-coated NC membrane and absorbent paper. To detect *Brucella* antibodies, the sample was deposited on the detection cassette. Following this, the cover body was placed on the cassette. Thus, the analyte was first fixed in the test zone and then bound to SPA-AuNPs, thereby minimizing false negatives. When *Brucella* antibodies were present in the sample, SPA-AuNPs bound to the anti-*Brucella* antibodies and IgGs, generating a color within 3 min. Similar to AuNP-based LFIA, colloidal AgNPs were used as colored NPs for the detection of staphylococcal enterotoxin B (SEB) in LFIA (Figure 5A) [65]. Using this strip, as low as 0.5 ppm of SEB was visually detected within 15 min, with the linear range being 0 to 2 ppm. Colloidal carbon was used for the development of LIFA strips for detecting *Mycoplasma bovis* [79]. Colloidal carbon has some advantages, such as simple conjugation and stability. Importantly, its bright-colored readout (black line on the white strip) leads to a high signal–background contrast, enhancing sensitivity [76,79]. In addition, the sensitivity could be enhanced by combining Au or Ag growth-mediated signal amplification methods, in which the in situ growth of NPs increased their size, thereby strengthening the signal readout [66,70]. NH_2_OH·HCl was used as a signal enhancer; in this method, AuNPs acted as catalysts for the reduction reaction between HAuCl_4_ and NH_2_OH·HCl, generating more AuNPs. The generated AuNPs were deposited on the test zone of the strip or covered the surface of the initially added AuNPs to form larger-sized AuNPs, providing a signal amplification effect. This signal amplification strategy combined with LFA exhibited 100-fold-enhanced sensitivity and could detect 10^4^ CFU/mL of *S. enteritidis*.

Concentrated NPs can also act as color generation factors because of these catalytic activities. NP-mediated chemical reactions can amplify visual signals [70,71,72]. NPs can interact with the substrate as signal amplifiers. Consequently, the substrate is converted into colorful chemicals by the catalytic activity of NPs. For example, a recent study used a combination of two methods for cascade signal amplification: the first method involved the use of NH_2_OH·HCl and hydroquinone for in situ AuNP growth and the second method involved the use of nanozyme-mediated catalytic deposition (Figure 5B) [70]. Similar to the use of NH_2_OH·HCl, the use of hydroquinone increased the size of the initially added AuNPs on the strip. The enlarged AuNPs had high catalytic activity and could oxidize the chromogen 3-amino-9-ethylcarbazole, resulting in the deposition of a colored product on the surface of AuNPs. This strip had a detection limit of 1.25 × 10^1^ CFU/mL for *E. coli* O157:H7. Pd-Pt NPs were also used for signal amplification to detect *E. coli* O157:H7 [71]. Pd-Pt NPs with peroxidase-like activity were conjugated with anti-*E. coli* O157:H7 antibodies and deposited on the conjugation pad of the LFIA strip. After injecting a sample containing bacterial cells on the strip, TMB was added to another antibody-deposited test zone. The concentrated Pd-Pt NPs at the test zone could oxidize TMB in the presence of H_2_O_2_, producing a colored product and visual signal. Similarly, Pt-Au NPs with peroxidase-like activity were used to amplify the signal readout and to detect anti-*E. coli* O157:H7, exhibiting a detection limit of 1 × 10^2^ CFU/mL within 1 min. The catalytic activity of NPs resulted in a rapid response depending on the activity and enhanced the sensitivity of the assay. This system would, however, require an additional process, such as the addition of substrates and enzymatic components.

## 4. Conclusions

This review summarizes the recent advances in colorimetric sensing systems for the detection of bacterial contamination in food, water, and environmental sources. Emphasis was placed on the different factors affecting the signal readout and color changes of substrates. This review confirms that colorimetric sensing systems are a rapidly developing technology with advantages such as simple detection and operation, low cost, and ability to operate without an instrument or detector.

Despite these advantages, there is still room to improve the sensing performance of systems, and to achieve enhanced capability (Table 2). Ongoing efforts have been made in the development of methods with enhanced sensing performance. Sensitivity can be enhanced by using a dual-readout system and chemicals with various sensing properties [34,36]. The use of optimal chemicals and concentrations can improve the detection limit of systems [19]. A sensitive system could also be obtained using a combination of signal amplification methods, such as HCR, RPA, HAD, RCA, and CHA [56,57,65,69,81,82,83,84]. Moreover, the use of a 3D paper structure embedded with a chemical reaction-related reagent can make systems simple to use [31,32]. An automation device can be combined with sensing systems to allow the detection of multiple bacteria in a simple manner [22]. Recently, much effort has also been given to the fabrication of enzyme-like functional NPs and their application in the detection of bacterial contamination [45]. Unlike traditional NP aggregation-based methods, which are mostly used for signal visualization, the chemical reaction-catalyzing NP-based strategy is minimally affected by the interference of the complex components of actual samples, providing relatively correct signals [45]. This sensing system can quantitatively detect the levels of bacteria and toxins in a broad range using digital cameras, smartphones, or mobile devices to obtain the color intensity and conduct data processing [22,23,34,36,112,113].

With ongoing advances, we believe that colorimetric sensing systems will be considered a promising option for monitoring and detecting bacterial contamination in food, water, and environmental sources during production or manufacturing processes.

## Figures and Tables

**Table 1:** Colorimetric sensing systems for the detection of pathogenic bacteria and toxins ^a^.

Material	Receptor	Target	Linear Range	LoD	Assay Time	Real Sample to Be Tested	Driving Force of Color Change	Feature	References
AuNPs, magnetic beads	Monoclonal antibody, polyclonal antibody	*Listeria monocytogenes*	1.1 × 10^2^ CFU/mL–1.1 × 10^6^ CFU/mL	100 CFU/mL	30 min	Lettuce samples	Induction of pH change	Use of magnetic nanobeads modified with urease and monoclonal antibodies.	[19]
								Use of AuNPs modified with urease and polyclonal antibodies.	
								Use of BCP.	
AgNPs	Monoclonal antibody	*Salmonella typhimurium*	1 × 10^8^ CFU/mL–1 × 10^1^ CFU/mL	100 CFU/mL		Apple juice, lake water sample	Induction of pH change	Based on the competitive binding ability of urease and bacterial cells to PEI-functionalized AgNPs.	[20]
Ag ion	None	*S. typhimurium*	1 × 10^7^ CFU/mL–1 × 10^1^ CFU/mL	100 CFU/mL		Tap water	Induction of pH change	Based on the Ag-induced inhibition of urease activity and Ag ion utilization.	[21]
								Combined with electrochemical sensing.	
NPs	Aptamer	*Escherichia coli.*, *S. typhimurium*	1 × 10^5^ CFU/mL–1 × 10^1^ CFU/mL	1 CFU/mL	<1 h	Milk	Induction of pH change	Use of pH-responsive NPs made of phenolphthalein (PP) and thymolphthalein (TP) indicators.	[22]
								Combined with automated equipment.	
								Allows multiplexing detection.	
Filter paper	None	Bacteria	11.2 × 10^3^–1.12 × 10^6^ CFU/g (using BTB), 38.0 × 10^3^–1.12 × 10^6^ CFU/g (using BCP)	11.64 × 10^3^ CFU/g		Chicken and meat samples	Induction of pH change	Monitoring of bacterial contamination level using paper-based pH indicators, BTB, and BCP.	[23]
								Sensing of external pH change caused by volatile basic nitrogen generated from bacterial spoilage.	
								Use of RGB analysis software on a smartphone.	
Filter paper	None	*E. coli*, *E. coli* O157:H7, *L. monocytogenes*, *Vibrio vulnificus*	1 × 10^6^–1 × 10^8^ CFU/mL	10 CFU/mL	1 h	Milk	Chemical reaction between intracellular enzymes and their chromogenic substrates	One-step-based 3D paper sensor functionalized with lysing and oxidizing agents.	[31]
Filter paper	None	*E. coli*, *E. coli* O157:H7	1 × 10^6^–1 × 10^9^ CFU/mL	10 CFU/mL	<4 h	Milk	Chemical reaction between intracellular enzymes and their chromogenic substrates	Use of a multi-layered paper structure.	[32]
								Use of β-glucuronidase and β-galactosidase-based enzymatic reactions.	
None	None	*Staphylococcus aureus*, *E. coli*	2.6 × 10^2^–1.16 × 10^9^ CFU/mL (for *E. coli*), 9.75 × 10^2^–6 × 10^9^ CFU/mL (for *S. aureus*)	ND	2 h	Drinking water, milk	Redox reaction between the cell counting kit-8 (CCK-8) solution and dehydrogenase	Measurement of formazan generated from the reduction reaction between dehydrogenase and CCK-8 (containing WST-8 and 1-methoxy-5-methylphenazinium methyl sulfate).	[33]
None	None	*E. coli*	1 × 10^4^–1 × 10^9^ CFU/mL	1 × 10^4^ CFU/mL	1 h	Unfiltered tap water	Reduction reaction of *p*-benzoquinone by intracellular enzymes	Use of RGB analysis software on a smartphone for quantification.	[34]
Filter paper	None	*E. coli*, *S. aureus*, *Enterococcus faecalis*, *Streptococcus mutans*, *Salmonella pullorum*	1 × 10^4^–1 × 10^8^ CFU/mL	7.48 × 10^3^ CFU/mL (for *E. coli*) and 3.3 × 10^3^ CFU/mL (for *S. aureus*)	20 min		Inhibition of GOx activity by glucose uptake of bacterial cells	Use of starch–iodide doping paper as a substrate.	[35]
								Based on the conversion from iodide to iodine by H_2_O_2_ involving GOx-mediated glucose oxidation (causing color change of starch–iodine) and glucose uptake of bacterial cells (causing inhibition of color change of starch–iodine).	
Filter paper	None	*E. coli*	1 × 10^2^–1 × 10^6^ CFU/mL	44 CFU/mL		Tap water, degrease milk	Inhibition of color change of OPD via Cu^2+^ reduction by intracellular enzymes	Use of paper as a substrate.	[36]
								Based on the competitive reaction between the oxidation of OPD by Cu^2+^ (causing color change of OPD) and the reduction of Cu^2+^ by bacteria (causing inhibition of color change of OPD).	
								Use of RGB analysis software on a smartphone for quantification.	
								Allows dual-readout assay (colorimetry and fluorescence).	
ZnFe_2_O_4_/rGO	Aptamer	*S. typhimurium*	11–1.10 × 10^5^ CFU/mL	11 CFU/mL		None	Peroxidase-like catalytic reaction of the ZnFe_2_O_4_/rGO nanostructure	Shows highly stable catalytic activity at low pH (over 5.5) and high temperature (over 50 °C).	[37]
Cu_2_-rGO NPs	None	*Salmonella* spp.	1.93 × 10^1^–1.93 × 10^5^ CFU/mL	0.51 CFU/mL		Milk	Peroxidase-like catalytic reaction of GO	Use of dsDNA amplified via PCR from cells. Based on the competitive binding of bacterial dsDNA and Cu_2_-rGO NPs to TMB.	[38]
Graphitic-C_3_N_4_@Cu_2_O	Aptamer	*S. typhimurium*	1.5 × 10^1^–1.5 × 10^5^ CFU/mL	15 CFU/mL	6 min	Milk	Peroxidase-like catalytic reaction of the g-C_3_N_4_@Cu_2_O nanostructure	Based on the competitive binding of the aptamer and g-C_3_N_4_@Cu_2_O to TMB.	[39]
Fe_3_O_4_/Au magnetic nanocomposite	Antibody, aptamer	*S. aureus*	1 × 10^1^–1 × 10^6^ CFU/mL	10 CFU/mL		Pork, milk	Peroxidase-like catalytic reaction of AuNPs by H_2_O_2_ etching	Use of a magnetic nanocomposite consisting of a Fe_3_O_4_ core and an Au shell as a capture probe.	[40]
								Use of Apt–AuNPs as a signal amplifier.	
AuNPs, magnetic beads	Antibody	Brevotoxin B	0.1–150 ng/kg	0.076 ng/kg		Seafood sample	Peroxidase-based TMB oxidation reaction	Addition of Fe^2+^ for color signal amplification.	[41]
Magnetic beads	Antibody	Ochratoxin A	0.01–10 ng/mL	8.3 pg/mL	30 min (for color development)	Red wine sample	Enzyme-controlled Turnbull’s blue generation	Based on the formation or inhibition of Prussian blue from K_3_[Fe(CN)_6_] via GOx-catalyzed H_2_O_2_ production.	[42]
Aptamer@ BSA- AuNCs	Aptamer	*S. typhimurium*	1 × 10^1^–1 × 10^6^ CFU/mL	1 CFU/mL		Eggshell, Egg white	Peroxidase-like catalytic reaction of AuNCs	Based on the enhanced catalytic activity of a cell-bound nanostructure (cell-aptamer@BSA-AuNC composite).	[43]
MnO_2_-doped Fe_3_O_4_ NPs	None	*S. aureus*, *Vibrio parahaemolyticus*	1 × 10^1^–1 × 10^6^ CFU/mL	1 × 10^2^ CFU/mL		Lake water sample	Peroxidase-like catalytic reaction	Use of multifunctional NPs for recognition, absorption, and separation of the analyte.	[44]
								Exhibits the catalytic activity of TMB in the presence of oxygen in a solution without H_2_O_2_.	
AuNPs	4-MPBA	*E. coli*	1 × 10^4^–1 × 10^7^ CFU/mL	1.02 × 10^3^ CFU/mL	20 min	Drinking water	Salt-induced aggregation	Use of AuNPs functionalised with 4-MPBA, which binds to LPS and peptidoglycan existing on the surface of gram-negative and gram-positive bacterial cells, respectively.	[48]
								Use of RGB analysis software on a smartphone for quantification.	
AuNPs	Aptamer	*Shigella flexneri*	1 × 10^2^–1 × 10^6^ CFU/mL	80 CFU/mL	20 min	Salmon	Salt-induced aggregation	Use of aptamers that can bind to bacterial cells rather than AuNPs.	[49]
AuNPs, silica nanoparticles (SNPs)	Aptamer	Aflatoxin M1	300–75,000 ng/L	30 ng/L		Milk	Salt-induced AuNP aggregation	Salt-induced aggregation by releasing complementary strands from aptamer-modified SNPs in the presence of the target.	[50]
AuNPs, magnetic nanoparticles (MNPs)	Antibody	*V. parahaemolyticus*	1 × 10^1^–1 × 10^6^ CFU/mL	10 CFU/mL		Oyster	Mn^2+^-induced AuNP aggregation	Combination with the signal amplification method based on ascorbic acid-mediated Mn^2+^ reduction and a sandwich assay using IgG-MnO_2_ NPs and IgY-MNPs.	[51]
AuNPs	Chimeric phage	*E. coli*, *V. cholerae*, *Pseudomonas aeruginosa*, *Xanthomonas campestris*		1 × 10^2^ CFU/mL	<1 h	Sea water, tap water	AuNP aggregation	Use of thiolated chimeric phages that can bind to both bacterial cells and AuNPs	[52]
AuNPs	Chimeric phage	*P. aeruginosa*	1 × 10^1^–1 × 10^6^ CFU/mL	1 × 10^2^ CFU/mL	~30 min	Drinking water, non-fat bovine milk	AuNP aggregation	Detection of antibiotic resistance/susceptibility of bacterial cells	[53]
Dextran-coated AuNPs, MNPs	Antibody	*E. coli*	1 × 10^3^–1 × 10^6^ CFU/mL	41 CFU/mL	95 min	Milk	ConA-driven aggregation of dextran-coated AuNPs	Use of ConA with pH-regulated transformation ability of dimers/tetramers	[54]
AuNPs		Fumonisin B1 (FB1)	2–8 mg/kg	0.9 mg/kg		Corn	Hydrolyzed FB1-induced AgNP aggregation	Use of cysteamine-functionalised AuNPs (Cys-AuNPs). Need for NaOH treatment to obtain hydrolyzed FB1 with a high affinity towards Cys-AuNPs.	[55]
AuNPs	DNA	*S. aureus*	1–1 × 10^5^ CFU/mL	1 CFU/mL	15 min	CSF, urine, spit, serum	Enzyme-driven DNA walker-induced AgNP aggregation	Use of an exonuclease III-driven DNA walker system for signal amplification.	[56]
AuNPs, MNPs	Aptamer	*S. typhimurium*	1 × 10^2^–1 × 10^6^ CFU/mL	2.4 × 10^2^ CFU/mL		Milk	Catalytic hairpin assembly (CHA)-driven AuNP aggregation	Use of Y-shaped CHA for signal amplification.	[57]
AgNPs	Antibody	Staphylococcal enterotoxin B	0–2 ppm	0.5 ppm	15 min	Milk, honey	AgNP accumulation	Use of AgNP-based sandwich-type lateral flow immunoassay (LFIA).	[65]
AuNPs	Antibody	*S. enteritidis*	1 × 10^5^–1 × 10^8^ CFU/mL	1 × 10^4^ CFU/mL	20 min	Milk	AuNP accumulation	Use of LFIA.	[66]
								Use of a signal enhancer, HAuCl_4_ and NH_2_OH·HCl for in situ AuNP growth.	
AuNPs	Antibody	*V. parahaemolyticus*		4.66 × 10^5^ CFU/mL	2 h	Oyster hemolymph	AuNP accumulation	Use of a dipstick.	[67]
AuNPs	Aptamer	*S. typhimurium*, *E. coli* O157:H7, *S. aureus*		1 × 10^3^ CFU/mL for *S. typhimurium* and 1 × 10^4^ CFU/mL for *E. coli* O157:H7 and *S. aureus*	10 min	Milk, chicken, food	AuNP accumulation	Use of LFA.	[68]
AuNPs, MNPs	Aptamer	*V. parahaemolyticus*	1 × 10^3^–1 × 10^8^ CFU/mL	2.6 × 10^3^ CFU/mL	67 min	Shrimp	AuNP accumulation	Combination of HCR-mediated signal amplification methods.	[69]
AuNPs	Antibody	*E. coli* O157:H7	1.25 × 10^1^–1.25 × 10^5^ CFU/mL	1.25 × 10^1^ CFU/mL		Milk	AuNP accumulation	Use of LFA.	[70];
								Combination of two signal amplification strategies; use of a signal enhancer (hydroquinone) for in situ AuNP growth and nanozyme-mediated catalytic deposition.	
Pd-Pt NPs	Antibody	*E. coli* O157:H7	1 × 10^2^–1 × 10^6^ CFU/mL	0.87 × 10^2^ CFU/mL	10 min	Milk	Pd-Pt NP accumulation-driven catalytic reaction	Use of LFA.	[71]
								Signal readout by oxidised TMB through Pd-Pt NP-mediated catalytic reactions.	
Pt-Au NPs	Antibody	*E. coli* O157:H7	1 × 10^2^–1 × 10^8^ CFU/mL	1 × 10^2^ CFU/mL	1 min		Pt-Au NP accumulation-driven catalytic reaction	Use of LFA.	[72]
								Use of Pt-Au-mediated signal amplification.	

^a^ Abbreviations: LoD, limit of detection; AuNPs, gold nanoparticles; CFU, colony-forming unit; TMB, 3,3′,5,5′-tetramethylbenzidine; BTB, bromothymol blue; BCG, bromocresol green; BCP, bromocresol purple; RGB, red-green-blue; PCR, polymerase chain reaction; PEI, polyethylenimine; WST-8, 2-(2-methoxy-4-nitrophenyl)-3-(4-nitrophenyl)-5-(2,4-disulfophenyl)-*2H*-tetrazolium, monosodium salt; OPD, *o*-phenylenediamine; dsDNA, double-stranded DNA; GO, graphene oxide; rGO, reduced GO; BSA, bovine serum albumin; GOx, glucose oxidase; AuNCs, gold nanoclusters; 4-MPBA, 4-mercaptophenylboronic acid; LPS, lipopolysaccharide; CSF, cerebral spinal fluid; ConA, concanavalin A; HCR, hybridization chain reaction; ND, not determined.

**Table 2:** Challenges and strategies to improve the performance of colorimetric systems for the detection of bacterial contamination.

Challenge	Performance Improvement Strategy	Reference(s)
Sensitivity	Use of pH-responsive NPs Use of a dual-readout method with chromogens for fluorescence and visible color Use of dual-readout with redox-active molecules with electrochemical and optical properties Selection of optimal pH-responsive molecules and chromogens Combination of signal amplification methods Use of a signal enhancer	[22] [36] [34] [19] [56,57,69,81,82,83,84] [70,71,72]
Simple operation	Use of a regent-embedded multi-layered paper structure Use of Ag ion without fabrication and functionalization of NPs Combined with an automation device	[32] [21] [22]
Correct signal in complex real samples	Based on the catalytic activity of mimetic enzymes (functional NPs or nanostructure)	[37,38,39,40,41,42,43,44]
Multiplexing capability	Combined with an automation device Based on multiple chromogenic or cellular metabolic reactions	[22] [31]
Quantification and expansion of analyzed spectra	Use of RGB analysis software on a smartphone	[22,23,34,36]

## Data Availability

No new data were created in this study. Data sharing is thus not applicable to this article.

## References

[1] Chen J., Andler S.M., Goddard J.M., Nugen S.R., Rotello V.M. (2016). Integrating recognition elements with nanomaterials for bacteria sensing. Chem. Soc. Rev..

[2] Florentin A., Lizon J., Asensio E., Forin J., Rivier A. (2016). Water and surface microbiologic quality of point-of-use water filters: A comparative study. Am. J. Infect. Control.

[3] Lemarchand K., Lebaron P. (2003). Occurrence of *Salmonella* spp. and *Cryptosporidium* spp. in a French coastal watershed: Relationship with fecal indicators. FEMS Microbiol. Lett..

[4] Farrell M.L., Joyce A., Duane S., Fitzhenry K., Hooban B., Burke L.P., Morris D. (2021). Evaluating the potential for exposure to organisms of public health concern in naturally occurring bathing waters in Europe: A scoping review. Water Res..

[5] Centers for Disease Control (2019). Outbreak of *E. coli* Infections Linked to Romaine Lettuce, *E. coli* Infections, *E. coli*, CDC. https://www.cdc.gov/ecoli/2019/o157h7-11-19/.

[6] Nguendo-Yongsi H.B. (2011). Microbiological evaluation of drinking water in a sub-saharan urban community (Yaunde). Am. J. Biochem. Mol. Biol..

[7] Delaire C., Peletz R., Kumpel E., Kisiangani J., Bain R., Khus R. (2017). How much will it cost to monitor microbial drinking water quality in sub-Saharan Africa?. Environ. Sci. Technol..

[8] Schirone M., Visciano P., Tofalo R., Suzzi G. (2019). Editorial: Foodborne pathogens: Hygiene and safety. Front. Microbiol..

[9] Harris M., Alzua M.L., Osbert N., Pickering A. (2017). Community-level sanitation coverage more strongly associated with child growth and household drinking water quality than access to a private toilet in rural Mali. Environ. Sci. Technol..

[10] Du H., Wang X., Yang Q., Wu W. (2021). Quantum dot: Lightning invisible foodborne pathogens. Trends Food Sci. Technol..

[11] Li T., Ou G., Chen X., Li Z., Hu R., Li Y., Yang Y., Liu M. (2020). Naked-eye based point-of-care detection of *E. coli* O157: H7 by a signal-amplified microfluidic aptasensor. Anal. Chim. Acta..

[12] Castle L.M., Schuh D.A., Reynolds E.E., Furst A.L. (2021). Electrochemical sensors to detect bacterial foodborne pathogens. ACS Sens..

[13] Luo K., Kim H.-Y., Oh M.-H., Kim Y.-R. (2020). Paper-based lateral flow strip assay for the detection of foodborne pathogens: Principles, applications, technological challenges and opportunities. Crit. Rev. Food Sci. Nutr..

[14] Verma M.S., Rogowski J.L., Jones L., Gu F.X. (2015). Colorimetric biosensing of pathogens using gold nanoparticles. Biotechnol. Adv..

[15] Choi Y., Hwang J.H., Lee S.Y. (2018). Recent trends in nanomaterials-based colorimetric detection of pathogenic bacteria and viruses. Small Methods.

[16] Filik H., Avan A.A. (2022). Nanotechnology-based colorimetric approaches for pathogenic virus sensing: A review. Curr. Med. Chem..

[17] Nguyen Q.H., Kim M.I. (2020). Nanomaterial-mediated paper-based biosensors for colorimetric pathogen detection. Trends Analyt. Chem..

[18] Liu B., Zhuang J., Wei G. (2020). Recent advances in the design of colorimetric sensors for environmental monitoring. Environ. Sci. Nano.

[19] Chen Q., Huang F., Cai G., Wang M., Lin J. (2018). An optical biosensor using immunomagnetic separation, urease catalysis and pH indication for rapid and sensitive detection of *Listeria monocytogenes*. Sens. Actuators B-Chem..

[20] Singh P., Kakkar S., Bharti, Kumar R., Bhalla V. (2019). Rapid and sensitive colorimetric detection of pathogens based on silver-urease interactions. Chem. Commun..

[21] Kumar V., Chopra A., Bisht B., Bhalla V. (2020). Colorimetric and electrochemical detection of pathogens in water using silver ions as a unique probe. Sci. Rep..

[22] Yan C., Sun Y., Yao M., Jin X., Yang Q., Wu W. (2022). pH-responsive nanoparticles and automated detection apparatus for dual detection of pathogenic bacteria. Sens. Acturator B-Chem..

[23] Abo Dena A.S., Khalid S.A., Ghanem A.F., Shehata A.I., El-Sherbiny I.M. (2021). User-friendly lab-on-paper optical sensor for the rapid detection of bacterial spoilage in packaged meat products. RSC Adv..

[24] Huang Y., Ran X., Lin Y., Ren J., Qu X. (2015). Enzyme-regulated the changes of pH values for assembling a colorimetric and multistage interconnection logic network with multiple readouts. Anal. Chim. Acta.

[25] Jankowska D.A., Bannwarth M.B., Schulenburg C., Faccio G., Maniura-Weber K., Rossi R.M., Scherer L., Richter M., Boesel L.F. (2017). Simultaneous detection of pH value and glucose concentrations for wound monitoring applications. Biosens. Bioelectron..

[26] Song Y., Qu K., Zhao C., Ren J., Qu X. (2010). Graphene oxide: Intrinsic peroxidase catalytic activity and its application to glucose detection. Adv. Mater..

[27] Contin A., Frasca S., Vivekananthan J., Leimkühler S., Wollenberger U., Plumeré N., Schuhmann W. (2015). A pH responsive redox hydrogel for electrochemical detection of redox silent biocatalytic processes. Control of Hydrogel Solvation. Electroanalysis.

[28] Bidmanova S., Hlavacek A., Damborsky J., Prokop Z. (2012). Conjugation of 5(6)-carboxyfluorescein and 5(6)-carboxynaphthofluorescein with bovine serum albumin and their immobilization for optical pH sensing. Sens. Actuators B-Chem..

[29] Chen C.H., Yang K.L. (2013). A liquid crystal biosensor for detecting organophosphates through the localized pH changes induced by their hydrolytic products. Sens. Actuators B-Chem..

[30] Lei C., Dai H., Fu Y., Ying Y., Li Y. (2016). Colorimetric sensor array for thiols discrimination based on urease-metal ion pairs. Anal. Chem..

[31] Kim H.J., Kwon C., Lee B.S., Noh H. (2019). One-step sensing of foodborne pathogenic bacteria using a 3D paper-based device. Analyst.

[32] Kim H.J., Kwon C., Noh H. (2019). Paper-based diagnostic system facilitating *Escherichia coli* assessments by duplex coloration. ACS Sens..

[33] Yang X., Zhong Y., Wang D., Lu Z. (2021). A simple colorimetric method for viable bacteria detection based on cell counting Kit-8. Anal. Methods.

[34] Sun J., Warden A.R., Huang J., Wang W., Ding X. (2019). Colorimetric and electrochemical detection of *Escherichia coli* and antibiotic resistance based on a *p*-benzoquinone-mediated bioassay. Anal. Chem..

[35] Sun J., Huang J., Li Y., Lv J., Ding X. (2019). A simple and rapid colorimetric bacteria detection method based on bacterial inhibition of glucose oxidase-catalyzed reaction. Talanta.

[36] Wang C., Gao X., Wang S., Liu Y. (2020). A smartphone-integrated paper sensing system for fluorescent and colorimetric dual-channel detection of foodborne pathogenic bacteria. Anal. Bioanal. Chem..

[37] Wu S., Duan N., Qiu Y., Li J., Wang Z. (2017). Colorimetric aptasensor for the detection of *Salmonella enterica* serovar *typhimurium* using ZnFe_2_O_4_-reduced graphene oxide nanostructures as an effective peroxidase mimetics. Int. J. Food Microbiol..

[38] Wang L., Liao T., Zhou H., Huang Y.V., Chen P., Yang X., Chen X. (2021). Colorimetric method for *Salmonella* spp. detection based on peroxidase-like activity of Cu(II)-rGO nanoparticles and PCR. Anal. Biochem..

[39] Tarokh A., Pebdeni A.B., Othman H.O., Salehnia F., Hosseini M. (2021). Sensitive colorimetric aptasensor based on g-C_3_N_4_@Cu_2_O composites for detection of *Salmonella typhimurium* in food and water. Mikrochim. Acta..

[40] Yao S., Li J., Pang B., Wang X., Shi Y., Song X., Xu K., Wang J., Zhao C. (2020). Colorimetric immunoassay for rapid detection of *Staphylococcus aureus* based on etching-enhanced peroxidase-like catalytic activity of gold nanoparticles. Mikrochim. Acta..

[41] Lai W., Wei Q., Zhuang J., Lu M., Tang D. (2016). Fenton reaction-based colorimetric immunoassay for sensitive detection of brevetoxin B. Biosens. Bioelectron..

[42] Lai W., Guo J., Wu Q., Chen Y., Cai Q., Wu L., Wang S., Song J., Tang D. (2020). A novel colorimetric immunoassay based on enzyme-regulated instant generation of Turnbull’s blue for the sensitive determination of ochratoxin A. Analyst.

[43] Chen Q., Gao R., Jia L. (2021). Enhancement of the peroxidase-like activity of aptamers modified gold nanoclusters by bacteria for colorimetric detection of *Salmonella typhimurium*. Talanta.

[44] Liu Y., Zhao C., Zhao W., Zhang H., Yao S., Shi Y., Li J., Wang J. (2020). Multi-functional MnO_2_-doped Fe_3_O_4_ nanoparticles as an artificial enzyme for the colorimetric detection of bacteria. Anal. Bioanal. Chem..

[45] Wu J., Wang X., Wang Q., Lou Z., Li S., Zhu Y., Qin L., Wei H. (2019). Nanomaterials with enzyme-like characteristics (nanozymes): Next-generation artificial enzymes (II). Chem. Soc. Rev..

[46] Bhardwaj N., Bhardwaj S.K., Bhatt D., Lim D.K., Kim K.H., Deep A. (2019). Optical detection of waterborne pathogens using nanomaterials. TrAC Trends Anal. Chem..

[47] Wei H., Wang E. (2013). Nanomaterials with enzyme-like characteristics (nanozymes): Next-generation artificial enzymes. Chem. Soc. Rev..

[48] Huang J., Sun J., Warden A.R., Ding X. (2020). Colorimetric and photographic detection of bacteria in drinking water by using 4-mercaptophenylboronic acid functionalized AuNPs. Food Control.

[49] Feng J., Shen Q., Wu J., Dai Z., Wang Y. (2019). Naked-eyes detection of *Shigella flexneri* in food samples based on a novel gold nanoparticle-based colorimetric aptasensor. Food Control.

[50] Jalalian S.H., Lavaee P., Ramezani M., Danesh N.M., Alibolandi M., Abnous K., Taghdisi S.M. (2021). An optical aptasensor for aflatoxin M1 detection based on target-induced protection of gold nanoparticles against salt-induced aggregation and silica nanoparticles. Spectrochim. Acta A Mol. Biomol. Spectrosc..

[51] Fu K., Zheng Y., Li J., Liu Y., Pang B., Song X., Xu K., Wang J., Zhao C. (2018). Colorimetric immunoassay for rapid detection of *Vibrio parahemolyticus* based on Mn^2+^ mediates the assembly of gold nanoparticles. J. Agric. Food Chem..

[52] Peng H., Chen I.A. (2019). Rapid colorimetric detection of bacterial species through the capture of gold nanoparticles by chimeric phages. ACS Nano.

[53] Peng H., Borg R.E., Nguyen A.B.N., Chen I.A. (2020). Chimeric phage nanoparticles for rapid characterization of bacterial pathogens, detection in complex biological samples and determination of antibiotic sensitivity. ACS Sens..

[54] Xu X., Yuan Y., Hu G., Wang X., Qi P., Wang Z., Wang Q., Wang X., Fu Y., Li Y. (2017). Exploiting pH-regulated dimer-tetramer transformation of concanavalin A to develop colorimetric biosensing of bacteria. Sci. Rep..

[55] Chotchuang T., Cheewasedtham W., Jayeoye T.J., Rujiralai T. (2019). Colorimetric determination of fumonisin B1 based on the aggregation of cysteamine-functionalized gold nanoparticles induced by a product of its hydrolysis. Mikrochim. Acta.

[56] Yang H., Xiao M., Lai W., Wan Y., Li L., Pei H. (2020). Stochastic DNA dual-walkers for ultrafast colorimetric bacteria detection. Anal. Chem..

[57] Chen S., Zong X., Zheng J., Zhang J., Zhou M., Chen Q., Man C., Jiang Y. (2021). A colorimetric strategy based on aptamer-catalyzed hairpin assembly for the on-site detection of *Salmonella typhimurium* in milk. Foods.

[58] Albanese A., Chan W.C. (2011). Effect of gold nanoparticle aggregation on cell uptake and toxicity. ACS Nano.

[59] Thuy Nguyen T.T., Han O.A., Lim E.B., Haam S., Park J.S., Lee S.W. (2021). The effect of pH and transition metal ions on cysteine-assisted gold aggregation for a distinct colorimetric response. RSC Adv..

[60] Dodero G., De Michieli L., Cavalleri O., Rolandi R., Oliveri L., Daccà A., Parodi R. (2000). L-Cysteine chemisorption on gold: An XPS and STM study. Colloids Surf. A.

[61] Aryal S., Remant B.K.C., Dharmaraj N., Bhattarai N., Kim C.H., Kim H.Y. (2006). Spectroscopic identification of S-Au interaction in cysteine capped gold nanoparticles. Spectrochim. Acta A Mol. Biomol. Spectrosc..

[62] Kreibig U., Genzel L. (1985). Optical absorption of small metallic particles. Surf. Sci..

[63] Brovko L.Y., Anany H., Griffiths M.W. (2012). Bacteriophages for detection and control of bacterial pathogens in food and food-processing environment. Adv. Food Nutr. Res..

[64] Yang X., Wisuthiphaet N., Young G.M., Nitin N. (2020). Rapid detection of *Escherichia coli* using bacteriophage-induced lysis and image analysis. PLoS ONE.

[65] Wu K.H., Huang W.C., Shyu R.H., Chang S.C. (2020). Silver nanoparticle-base lateral flow immunoassay for rapid detection of *Staphylococcal* enterotoxin B in milk and honey. J. Inorg. Biochem..

[66] Bu T., Huang Q., Yan L., Huang L., Zhang M., Yang Q., Yang B., Wang J., Zhang D. (2018). Ultra technically-simple and sensitive detection for *Salmonella enteritidis* by immunochromatographic assay based on gold growth. Food Control.

[67] Rodriguez-Quijada C., Lyons C., Santamaria C., Quinn S., Tlusty M.F., Shiaris M., Hamad-Schifferli K. (2020). Optimization of paper-based nanoparticle immunoassays for direct detection of the bacterial pathogen *V. parahaemolyticus* in oyster hemolymph. Anal. Methods.

[68] Lu C., Gao X., Chen Y., Ren J., Liu C. (2020). Aptamer-based lateral flow test strip for the simultaneous detection of *Salmonella typhimurium*, *Escherichia coli* O157:H7 and *Staphylococcus aureus*. Anal. Lett..

[69] Ying N., Wang Y., Song X., Yang L., Qin B., Wu Y., Fang W. (2021). Lateral flow colorimetric biosensor for detection of *Vibrio parahaemolyticus* based on hybridization chain reaction and aptamer. Mikrochim. Acta.

[70] Fu J., Zhou Y., Huang X., Zhang W., Wu Y., Fang H., Zhang C., Xiong Y. (2020). Dramatically enhanced immunochromatographic assay using cascade signal amplification for ultrasensitive detection of *Escherichia coli* O157:H7 in milk. J. Agric. Food Chem..

[71] Han J., Zhang L., Hu L., Xing K., Lu X., Huang Y., Zhang J., Lai W., Chen T. (2018). Nanozyme-based lateral flow assay for the sensitive detection of *Escherichia coli* O157:H7 in milk. J. Dairy Sci..

[72] Jiang T., Song Y., Wei T., Li H., Du D., Zhu M.J., Lin Y. (2016). Sensitive detection of *Escherichia coli* O157: H7 using Pt-Au bimetal nanoparticles with peroxidase-like amplification. Biosens. Bioelectron..

[73] Park J., Shin J.H., Park J.-K. (2016). Pressed paper-based dipstick for detection of foodborne pathogens with multistep reactions. Anal. Chem..

[74] Prakash C., Kumar B., Singh R.P., Singh P., Shrinet G., Das A., Ashmi M., Abhishek Singh K.P., Singh M.K., Gupta V.K. (2021). Development and evaluation of a gold nanoparticle based lateral flow assay (LFA) strip test for detection of *Brucella* spp.. J. Microbiol. Methods.

[75] Huang Q. (2021). Simultaneous quantitative analysis of *Listeria monocytogenes* and *Staphylococcus aureus* based on antibiotic-introduced lateral flow immunoassay. Anal. Methods.

[76] Porras J.C., Bernuz M., Marfa J., Pallares-Rusiñol A., Martí M., Pividori M.I. (2021). Comparative study of gold and carbon nanoparticles in nucleic acid lateral flow assay. Nanomaterials.

[77] Wu K.H., Huang W.C., Chang S.C., Shyu R.H. (2022). Colloidal silver-based lateral flow immunoassay for detection of profenofos pesticide residue in vegetables. RSC Adv..

[78] Liang P., Guo Q., Zhao T., Wen C.Y., Tian Z., Shang Y., Xing J., Jiang Y., Zeng J. (2022). Ag nanoparticles with ultrathin Au shell-based lateral flow immunoassay for colorimetric and SERS dual-mode detection of SARS-CoV-2 IgG. Anal. Chem..

[79] Shi F., Zhao Y., Sun Y., Chen C. (2020). Development and application of a colloidal carbon test strip for the detection of antibodies against *Mycoplasma bovis*. World J. Microbiol. Biotechnol..

[80] Li C.M., Li Y.F., Wang J., Huang C.Z. (2010). Optical investigations on ATP-induced aggregation of positive-charged gold nanoparticles. Talanta.

[81] Du X.J., Zhou T.J., Li P., Wang S. (2017). A rapid *Salmonella* detection method involving thermophilic helicase-dependent amplification and a lateral flow assay. Mol. Cell. Probes.

[82] Özay B., McCalla E.S. (2021). A review of reaction enhancement strategies for isothermal nucleic acid amplification reactions. Sens. Actuator Rep..

[83] Liu J., Zhang Y., Xie H., Zhao L., Zheng L., Ye H. (2019). Applications of catalytic hairpin assembly reaction in biosensing. Small.

[84] Wu Y., Fu C., Shi W., Chen J. (2021). Recent advances in catalytic hairpin assembly signal amplification-based sensing strategies for microRNA detection. Talanta.

[85] Chen Q., Lin J., Gan C., Wang Y., Wang D., Xiong Y., Lai W., Li Y., Wang M. (2015). A sensitive impedance biosensor based on immunomagnetic separation and urease catalysis for rapid detection of *Listeria monocytogenes* using an immobilization-free interdigitated array microelectrode. Biosens. Bioelectron..

[86] Chen Q., Wang D., Cai G., Xiong Y., Li Y., Wang M., Huo H., Lin J. (2016). Fast and sensitive detection of foodborne pathogen using electrochemical impedance analysis, urease catalysis and microfluidics. Biosens. Bioelectron..

[87] Du N., Chen M., Liu Z., Sheng L., Xu H., Chen S. (2012). Kinetics and mechanism of jack bean urease inhibition by Hg^2+^. Chem. Cent. J..

[88] Ren R., Cai G., Yu Z., Zeng Y., Tang D. (2018). Metal-polydopamine framework: An innovative signal-generation tag for colorimetric immunoassay. Anal. Chem..

[89] Jiao L., Yan H., Xu W., Wu Y., Gu W., Li H., Du D., Lin Y., Zhu C. (2019). Self-assembly of all-inclusive allochroic nanoparticles for the improved ELISA. Anal. Chem..

[90] Courbat J., Briand D., Damon-Lacoste J., Wöllenstein J., de Rooij N.F. (2009). Evaluation of pH indicator-based colorimetric films for ammonia detection using optical waveguides. Sens. Actuators B Chem..

[91] Xu W., Jiao L., Ye H., Guo Z., Wu Y., Yan H., Gu W., Du D., Lin Y., Zhu C. (2020). pH-responsive allochroic nanoparticles for the multicolor detection of breast cancer biomarkers. Biosens. Bioelectron..

[92] Hodgkinson V., Petris M.J. (2012). Copper homeostasis at the host-pathogen interface. J. Biol. Chem..

[93] Rensing C., Grass G. (2003). *Escherichia coli* mechanisms of copper homeostasis in a changing environment. FEMS Microbiol. Rev..

[94] Zhang L., Li M., Qin Y., Chu Z., Zhao S. (2014). A Convenient label free colorimetric assay for pyrophosphatase activity based on a pyrophosphate-inhibited Cu^2+^-ABTS-H_2_O_2_ reaction. Analyst.

[95] Liang L., Huang Y., Liu W., Zuo W., Ye F., Zhao S. (2020). Colorimetric detection of salicylic acid in aspirin using MIL-53(Fe) nanozyme. Front. Chem..

[96] Zhang L., Hou Y., Guo X., Liu W., Lv C., Zhang C., Jin Y., Li B. (2020). Fe(III) bipyridyl or phenanthroline complexes with oxidase-like activity for sensitive colorimetric detection of glutathione. Luminescence.

[97] Gao L., Zhuang J., Nie L., Zhang J., Zhang Y., Gu N., Wang T., Feng J., Yang D., Perrett S. (2007). Intrinsic peroxidase-like activity of ferromagnetic nanoparticles. Nat. Nanotechnol..

[98] Jia H., Yang D., Han X., Cai J., Liu H., He W. (2016). Peroxidase-like activity of the Co_3_O_4_ nanoparticles used for biodetection and evaluation of antioxidant behavior. Nanoscale.

[99] Chen W., Fang X., Li H., Cao H., Kong J. (2017). DNA-mediated inhibition of peroxidase-like activities on platinum nanoparticles for simple and rapid colorimetric detection of nucleic acids. Biosens. Bioelectron..

[100] Li J., Liu W., Wu X., Gao X. (2015). Mechanism of pH-switchable peroxidase and catalase-like activities of gold, silver, platinum and palladium. Biomaterials.

[101] Wang S., Cazelles R., Liao W.C., Vázquez-González M., Zoabi A., Abu-Reziq R., Willner I. (2017). Mimicking horseradish peroxidase and NADH peroxidase by heterogeneous Cu^2+^-modified graphene oxide nanoparticles. Nano Lett..

[102] Lai W., Tang D., Zhuang J., Chen G., Yang H. (2014). Magnetic bead-based enzyme-chromogenic substrate system for ultrasensitive colorimetric immunoassay accompanying cascade reaction for enzymatic formation of squaric acid-iron(III) chelate. Anal. Chem..

[103] Zhang L., Du J. (2016). Selective sensing of submicromolar iron(III) with 3,3′,5,5′-tetramethylbenzidine as a chromogenic probe. Spectrochim. Acta A Mol. Biomol. Spectrosc..

[104] Zheng A., Zhang X., Gao J., Liu X., Liu J. (2016). Peroxidase-like catalytic activity of copper ions and its application for highly sensitive detection of glypican-3. Anal. Chim. Acta.

[105] Zhu N., Zou Y., Huang M., Dong S., Wu X., Liang G., Han Z., Zhang Z. (2018). A sensitive, colorimetric immunosensor based on Cu-MOFs and HRP for detection of dibutyl phthalate in environmental and food samples. Talanta.

[106] Wang Q., Pang H., Dong Y., Chi Y., Fu F. (2018). Colorimetric determination of glutathione by using a nanohybrid composed of manganese dioxide and carbon dots. Mikrochim. Acta.

[107] Yuan Q., He J., Niu Y., Chen J., Zhao Y., Zhang Y., Yu C. (2018). Sandwich-type biosensor for the detection of alpha2, 3-sialylated glycans based on fullerene-palladium-platinum alloy and 4-mercaptophenylboronic acid nanoparticle hybrids coupled with Au-methylene blue-MAL signal amplification. Biosens. Bioelectron..

[108] Bryła M., Roszko M., Szymczyk K., Jędrzejczak R., Obiedziński M.W. (2016). Fumonisins and their masked forms in maize products. Food Control.

[109] Voss K.A., Smith G.W., Haschek W.M. (2007). Fumonisins: Toxicokinetics, mechanism of action and toxicity. Anim. Feed Sci. Technol..

[110] Li Y.S., Zhou Y., Lu S.Y., Guo D.J., Ren H.L., Meng X.M., Zhi B.H., Lin C., Wang Z., Li X.B. (2012). Development of a one-step test strip for rapid screening of fumonisins B1, B2 and B3 in maize. Food Control.

[111] Shi F., Sun Y., Wu Y., Zhu M., Feng D., Zhang R., Peng L., Chen C. (2020). A novel, rapid and simple method for detecting brucellosis based on rapid vertical flow technology. J. Appl. Microbiol..

[112] Morbioli G.G., Mazzu-Nascimento T., Stockton A.M., Carrilho E. (2017). Technical aspects and challenges of colorimetric detection with microfluidic paper-based analytical devices (μPADs)—A review. Anal. Chim. Acta.

[113] Kanchi S., Sabela M.I., Mdluli P.S., Inamuddin, Bisetty K. (2018). Smartphone based bioanalytical and diagnosis applications: A review. Biosens. Bioelectron..

